# Urban ecosystem services research in Russia: Systematic review on the state of the art

**DOI:** 10.1007/s13280-024-02102-8

**Published:** 2024-11-23

**Authors:** Diana Dushkova, Anastasia Konstantinova, Victor Matasov, Dara Gaeva, Elvira Dovletyarova, Mina Taherkhani

**Affiliations:** 1 Department of Conservation Biology and Social-Ecological Systems; Department of Urban and Environmental Sociology, Helmholtz-Centre for Environmental Research (UFZ), Permoser str. 15, 04318 Leipzig, Germany; 2 Agrarian and Technological Institute, Peoples Friendship University of Russia (RUDN University), Miklukho-Maklaya str., 6, Moscow, Russia 117198; 3 Faculty of Geography and Geoinformation Technology, Higher School of Economics (HSE University), 11 Pokrovsky Boulevard, Moscow, Russia 109028; 4 Immanuel Kant Baltic Federal University, A. Nevskogo 14, Kaliningrad, Russia 236016

**Keywords:** Ecosystem services, Environment, Russia, Sustainable development, Urban blue-green infrastructure, Urban ecosystems

## Abstract

Research on urban ecosystem services (ES) covers many regions globally, yet significant gaps remain in several areas, including Russia. Furthermore, the number of publications on Russian ES is still very low, and most of them focus on ES assessment on the national level which results in an incomplete understanding of ES provision in Russian cities. To fill this gap, 197 publications on urban ES have been systematically reviewed. Those mostly included research on urban ES in Russia as well as in other cities of the world that represented case studies within international collaborations of Russian scientists with other partners. The paper presents the main aspects of the ES concept adoption and operationalizing in the Russian scientific discourse, by providing an overview of the current research on the relationships between humans and urban ecosystems. Spatially, the majority of studies focused on large cities; herewith field surveys, statistical analysis, expert evaluations, and mapping were often used as the main research methods. Four key challenge areas have been revealed: anthropogenic pressure on soils and vegetation, urban green spaces (UGS) and their quantity and quality for ES provision, the value of ES assessment for urban planning, and payments for ES. To address these challenges, future research should focus on the role of urban ES for city resilience and sustainability, ecosystem disservices, rural–urban interactions, broader testing and validation of ES mapping and assessment as well as better integration of ES in economic, regulatory and compensation mechanisms.

**Supplementary Information:**

The online version contains supplementary material available at 10.1007/s13280-024-02102-8.

## Introduction

From its appearance in the 1990s (de Groot 1992; Costanza et al. 1997; Daily 1997; de Groot 2002; de Groot et al. 2010), the concept of ES and its practical application received significant attention (MEA 2005; TEEB 2008, 2010; Haines-Young and Potschin-Young 2018). And yet it still remains the primary focus of integrated inter- and transdisciplinary research (Costanza 2008; Maes et al. 2011; Burkhard et al. 2012; Haines-Young and Potschin 2013; Haase et al. 2014; Haines-Young and Potschin 2018). This is mainly due to the fact that mapping and valuing ES could significantly enhance stewardship and assist in uncovering the hidden economic value of natural capital and environmental quality (Jiang et al. 2021). Moreover, it encourages decision-makers to adopt more scientifically informed strategies for sustainable development. ES are defined as “…the benefits people obtain from ecosystems, both natural and managed” (MEA 2005) in the form of the natural resources (e.g. food, water, etc.) and services (e.g. climate regulation, resisting the spread of infectious diseases, etc.). As a matter of fact, ES allow to link biophysical aspects of ecosystems with human benefits (de Groot et al. 2010) which first of all include supporting, ensuring, and sustaining human livelihood and quality of life (Foley et al. 2005; Wallace 2007; Gomez-Baggethun et al. 2010; Haase et al. 2014; Remme et al. 2021). Beyond its essential role in biodiversity conservation and enhancing environmental quality, the concept of ES also greatly contributes to raising public awareness on the importance and value of nature (Daily et al. 2009; Haase et al. 2014; TEEB DE 2016; Costanza 2020; Jiang et al. 2021). In doing so, it supports decision-makers in implementing more scientifically evident sustainable development strategies and environmental policies (TEEB DE 2016). Therefore, building sustainable economies and recognizing the value of nature to drive policy advancements in maintaining ES are the main goals of the concept. Thus, to advance towards the aforementioned goals, an increasing number of countries are engaging in national TEEB processes at various levels, including national, sub-national, and corporate initiatives (TEEB 2013; TEEB RU 2 2020; Bukvareva and Grunewald 2020).

There is a significant number of activities underway throughout the EU concerning the mapping, assessment, and evaluation of ecosystems and their services (e.g. MEA 2005; TEEB 2010; CBD 2011; activities of the IPBES for integration of the ES concept into policy agendas; activities of the ES-Partnership; partnerships and developing of ES assessment tools within the Natural Capital Project; the SEEA EA as a UN statistical standard to integrate ES into national accounting processes).

Since the release of TEEB at the UN-CBD meeting in Nagoya, Japan in 2010, numerous countries have begun or already completed the national TEEB studies. This trend is also observed in Russia (e.g. TEEB-Russia 1, 2013–2016; TEEB-Russia 2, 2018–2019); see Grunewald et al. 2014a; Bukvareva and Zamolodchikov 2018; Bukvareva and Grunewald 2020; Bukvareva and Sviridova 2020; Klimanova 2021). Some of TEEB Russia are presented in the relevant sections of the 5th National Report on the Conservation of Biodiversity in the Russian Federation (Minprirody 2015) and the State reports “On the State and Protection of the Environment of the Russian Federation” (Minprirody 2021, 2023). Even though these reports recognize the importance of ecosystems, their services and economic assessment of ES, they also point out that Russia still does not actively use this approach in nature conservation. The Strategic Environmental Assessments of mega-projects set as a priority for the future to include the use of economic mechanisms in biodiversity conservation as well as economic assessment of ES in nature protection (Minprirody 2023).

Numerous research initiatives on biodiversity conservation have been undertaken in Russia to evaluate ES (Bukvareva et al. 2015; Nevedrov and Protsenko 2018; Bukvareva et al. 2019a, 2019b; Matasov et al. 2020; Aleksandriiskaia 2021; Illarionova et al. 2021). However, there is still a lack of interest among state representatives and decision-makers on this matter (Bobylev et al. 2019; Klimanova 2021; Konstantinova et al. 2023). For example, efforts have been made to assess the global significance of Russia's ecosystems using ecological and monetary parameters as well as to implement evaluations at the national and regional levels (Bobylev and Zakharov 2009; Bobylev 2014, 2015; Bukvareva et al. 2019b; Medvedeva 2020; Pashkevich et al. 2020; Semenyuk et al. 2020; TEEB Russia 1/2016, TEEB Russia 2/2020). Specific projects have focused on ES in regions like Kamchatka, the Altai, Lake Baikal, and the Lower Volga River (Zavadskaya et al. 2017; Bondarenko et al. 2019; Minprirody 2021). Also, a handbook on “Economy of the Biodiversity Conservation” (Tishkov et al. 2002) was produced. To sum up, theoretical frameworks and practical regional experiences for evaluating ES already exist (Grunewald et al. 2014a, b; Bukvareva et al. 2015; Klimanova et al. 2023). Still, Russia has limited involvement in the Ecosystem Services Partnership organization (ESP), and Russian researchers have minimal presence in the scientific literature on global ES. One of the reasons is that Russian authors mostly publish in Russian due to the lack of funding for open-access publications. Thus, the number of English language publications in the international literature on ES in Russia is still limited (Hegetschweiler et al. 2017; Acharya et al. 2019). So, aligning the ES concept with Russian scientific traditions could offer significant contributions to the studies of ES on a broader international scale (Bastian et al. 2015; Bukvareva et al. 2021; Klimanova et al. 2023).

As we realized, a vast literature on ES research in Russia deals extensively with either (a) the environmental changes (Tykkylainen et al. 2008; AMAP 2010; Krasovskaya and Evseev 2013; Bukvareva et al. 2019a, 2019b; Evseev et al. 2022) or (b) a measuring of ES on the national level as a part of natural capital (Bobylev and Zakharov 2009; Bobylev et al. 2012; Grunewald et al. 2014a, 2014b; Bastian et al. 2015; Bukvareva et al. 2015; Tishkov et al. 2019; Malygina et al. 2021). Nevertheless, research on the assessment of ES in urban areas of Russia barely exists (except for the studies of Sulkarnaeva 2018; Klimanova 2021; Klimanova et al. 2023). In particular, Russian urban areas still lack the proper focus of research on ES from different perspectives such as demand, supply, and capacity of ecosystems to provide the services. It is mostly due to the complexity of the factors (e.g. economic, industrial, socio-demographic, and environmental changes) that influence the urban ecosystems. Although the concept of ES has been advocated in international scientific community and political circles (MA 2005; Müller et al. 2010; TEEB 2010; Seppelt et al. 2011; Bastian et al. 2012; Angelstam et al. 2013; Elmqvist et al. 2013; Haase et al. 2014; Howe et al. 2014; Escobedo et al. 2019), empirical results from its application in an urban context of Russia are still insufficient, which in turn seems to be a white spot on the map of studies on urban ES (Brzoska and Spāǵe 2020; Pinto et al. 2022).

Against this background, in our systematic literature review, we aimed to analyse the variety of publications on urban ES in Russia to respond to the main research question: what is the current state of research on urban ES in Russia? For this purpose, the following subquestions were focused on:

- What ES were analysed and assessed in the different case study regions/cities of Russia (possible grouping of different research approaches by identifying the main research areas, methods, and goals)?
- What are the pathways in urban ES research in Russia (e.g. how the concept of ES was adapted to the reality of the Russian scientific context and what are the main directions of its further development)?

While structuring our review around these research questions, the key features of urban ES in Russia are examined to establish a foundation for further research. The paper consists of the following sections: methodology presents a general approach used in the study and gives an overview of the materials (data sources) and methods used, explaining literature review steps. The results section provides a closer look at the ES-related terms used in Russian scientific literature and specifies general patterns in the reviewed studies. In particular, the analysis of case study research contains two domains: the spatial distributions and the thematic analyses of these studies. Finally, the qualitative analysis of the most frequently cited studies is provided to summarize and systematize the findings which are also critically reflected in the Discussion section. This paper is one of the first in a series of review papers that hopefully will support learning among scientists, practitioners, and decision-makers with regard to research and management of urban ES in Russia.

## Materials and methods

### Place-based context: an overview of ES in Russia and key related definitions

ES of Russia present an important research field within the intertwined complexities of land cover and land use change, globalization, urbanization, industry restructuring, and post-Soviet transition. Russia, and in particular Russian North (making up to 66% of its territories or 10 million sq. km with a population of 9.4 million people (Fauzer et al. 2023)) has more intact ecosystems than any other country/nation on the planet (Dushkova et al. 2016; Grunewald et al. 2014b; Bukvareva et al. 2019a; Danilov-Danilyan et al. 2023). Additionally, more than half of Russian territory is not burdened by the presence of either man or technology in terms of significant traces of human activity (e.g. settlements, roads, water reservoirs, power transmission lines, cultivated fields, etc.). Only 3–4% of Europe’s territory belongs to that category (Bukvareva et al. 2021). Russia is home to a great diversity of natural capital (NC) and ecosystems that are suppliers of ES. The Russian territories make an important contribution to the Earth’s global biodiversity, climate stability, global carbon sequestration, and storage (net carbon sinks). Moreover, it plays a fundamental role in the preservation of the ethnic and cultural diversity of the indigenous population and their traditional land use (Strategy 2020; Evseev et al. 2022). Russia contributes strategically to the carbon deposition and sequestration provided by forests, wetlands, and peatlands covering, respectively, 45% and 22% of the country's territory (Zamolodchikov et al. 2011; Klyuev 2015; Schepaschenko et al. 2021; Danilov-Danilyan et al. 2023). The carbon content of the Russian wetlands takes up between 20% and 50% of the total worldwide amount of peat (Smith et al. 2004). Russia's permafrost contains the largest reservoir of carbon in terrestrial ecosystems, spanning approximately 11 million sq. km (Bukvareva et al. 2015). Hydrocarbons and minerals exist in significant quantities, possessing strategic importance on a global scale. Additionally, there are abundant fisheries resources and expansive areas suitable for domestic reindeer husbandry (Strategy 2020; Abakumov et al. 2022).

It should be noted that Russia has a high level of urbanization: 75% of its population lives in 1125 cities (2024) where the largest share of the country's GDP is created, and 16 of these cities have a population of more than 1 million people.^1^ Moreover, this trend tends to continue (Klimanova et al. 2021) with the increasing negative impact of urbanization on urban ecosystems and people that, as a consequence, reduces the efficiency of ecosystem services and their capacity to ensure a healthy and comfortable urban environment. Considering this fact as well as the previously revealed lack of publications on the assessment of ES in urban areas of Russia (except Sulkarnaeva 2018; Klimanova 2021; Klimanova et al. 2023), it is highly important to further investigate urban ES provision, demand, and supply as well as the role of ES for the well-being of citizens and sustainable development of urban areas.

In this paper, *urban ES* are defined as the benefits that urban residents obtain from urban nature (both natural and semi-natural and man-made ecosystems) within the urban blue-green infrastructure (UBGI) (similar to the approaches of Breuste et al. 2013; Haase et al. 2014; Klimanova et al. 2021). Urban ES encompass all functions and processes of urban ecosystems that contribute to human well-being, either economically or in terms of quality of life.

Since in cities, green infrastructure (GI) is a key source of urban ES, its definition and use in the Russian scientific discourse are also included in the scope of this research. In Russian literature, the GI concept (rarely called BGI) is closely linked to the concepts of “urban ecological network or framework” (introduced by Vladimirov 1986) or “nature framework” (suggested by Rejmers 1990). Both concepts originally appeared as a mechanism of nature preservation and establishment of ecological zones in the city. However, the term “ecological network” still has no legal status in Russia, in comparison with the urban “network of green areas/urban green spaces” (UGS) that has been integrated into the first Soviet urban planning system (so-called General Plans/Genplans, see Dushkova et al. 2023) since the 1930s. From that time, Genplans became official documents established by the governments as a basis for urban planning, reconstruction, and development. Along with the network of UGS, Russian scientists and urban planners often use the term “green plantings” defined as “a complex of tree, shrub and herbaceous vegetation in a specific area”. Here it is important to note that GI includes more elements than the network of UGS, in terms of their volume, range, integrity, and connectivity. The term “blue-green infrastructure” has relatively recently appeared in Russian literature and is not mentioned in official regulatory documents (its alternatives are “the system of UGS” or “green zone of the city”). In our research, we refer to the definition of Klimanova et al. (2021, p.81) that UBGI includes “the internal ecological framework, namely recreational green areas (forest parks, parks, squares, boulevards, embankments), green areas performing sanitary and hygienic functions (sanitary protection zones, green space around hospitals, schools, kindergartens) as well as artificial and natural water bodies”. UBGI encompasses all unbuilt UGS which differ in their functionality, connectivity, and hierarchy (Klimanova et al. 2021).

### General approach

**Fig. 1:**
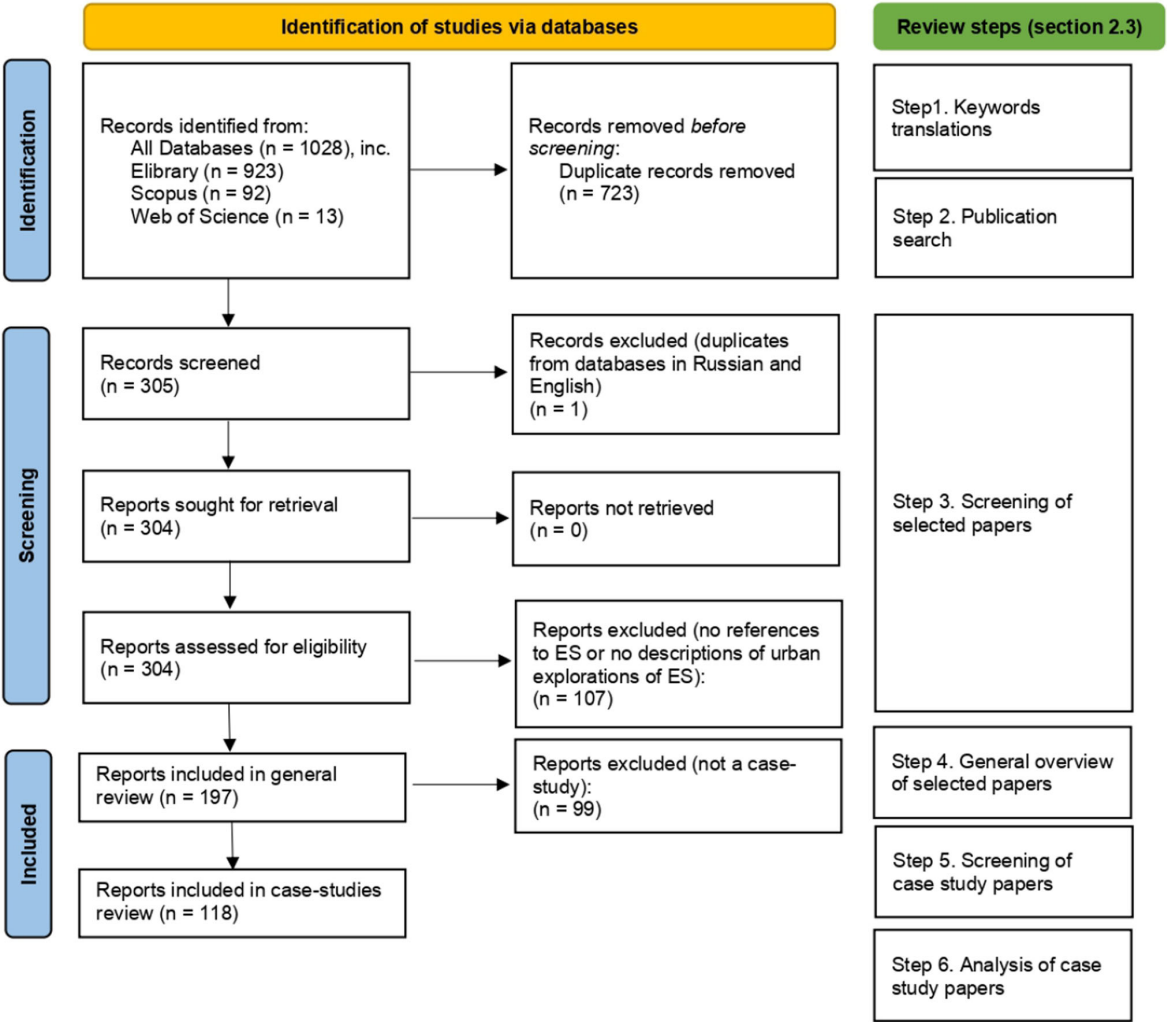
PRISMA flow diagram showing the steps of the methodology and the selection process used for the systematic literature review applied in this study

We focused on peer-reviewed articles published in international and national (Russian) scientific journals and studies from national reports. However, studies published in monographs, textbooks, and local planning documents are not a part of this review. For our systematic literature review, we used PRISMA protocol (Preferred Reporting Items for Systematic Reviews and meta Analyses) (Page et al. 2021). The flow diagram presenting the key steps of the methodology and the selection process is shown in Fig. 1. A general approach, including the steps and materials used, is described in detail in the next section. Additionally, key words translation (Russian equivalents for search) as a preliminary step was done before starting the identification of studies (step 1). After publication search (step 2), screening of selected papers (step 3), screening of reports selected for retrieval and assessed for eligibility (step 4), the sample of 118 scientific papers has been finally included in the review including their spatial and thematic analysis (step 6) (see Section "Materials (data sources)").

Our review considered articles published between 2006 and 2021 because relevant studies have been extensively published in overwhelming numbers since the beginning of 2006. This boom in environmental research was probably due to the increased attention paid to rapid and intensified urbanization, challenges related to climate change as well as appearance of the one of the key publications on ES—MEA (2005) which has increased related research, also in Russia. In addition, starting from that period, more research funding programmes have focused on how people interact with nature to improve their health and social well-being and how to enhance the health of ecosystems worldwide (e.g. funded under the Horizon 2020 ERA-NET COFUND scheme such as BiodivERsA, eLTER RI—European Long-Term Ecosystem, Critical Zone and Socio-ecological Research Infrastructure).

### Materials (data sources)

Our objective was to organize a systematic procedure of literature selection and analysis that should be replicable (as pointed out by Konijnendijk et al. 2013). In addition to ISI Web of Science©, and Scopus©, also major scientific Russian internet resources were used because not so many papers were published in English and not many Russian scientific journals were included in international databases. Thereby the data sources included nine Russian databases from different libraries, scientific areas, catalogues, etc. (Table 1).

**Table 1:** The main digital catalogues for searching study related papers in various Russian libraries

№	Name of the Russian Library	Online public access catalogue	Art of resources	Internet	Language
1	eLibrary—The Scientific Electronic Library (Hayчнaя элeктpoннaя библиoтeкa)	Catalogue of Journals	The main resource for searching for papers in scientific journals, also proceedings, books, etc	http://elibrary.ru	Russian
2	The Russian State Library—RSL (PГБ—Poccийcкaя гocyдapcтвeннaя библиoтeкa, former “Leninka”)	Integrated digital catalogue of RSL (Eдиный элeктpoнный кaтaлoг PГБ)	Books from 1831 onwards, dissertations, abstracts of dissertations (aвтopeфepaты диccepтaций), maps	http://aleph.rsl.ru/F/?func=file&file_name=find-a	Russian, English
3	Russian National Library, St.-Petersburg (PHБ – Poccийcкaя нaциoнaльнaя библиoтeкa)	Digital catalogue of RNL (Элeктpoнный кaтaлoг PHБ)	Books from 1708 onwards, abstracts of dissertations	http://www.nlr.ru/poisk/#1	Russian, English
4	Library for Natural Sciences of the Russian Academy of Science, Moscow (Библиoтeкa пo ecтecтвeнным нayкaм PAH)	Journals catalogue (Элeктpoнныe pecypcы – кaтaлoг жypнaлoв); book catalogue (Элeктpoнныe pecypcы – кaтaлoг книг)	Papers, books, abstracts/proceedings of conferences with the focus on natural sciences	http://www.benran.ru/Magazin/Catalog/Catalog.htm http://www.benran.ru/cbook_en/	Russian, English
5	State public scientific and technical library, Moscow (ГПHTБ – Гocyдapcтвeннaяпyбличнaянayчнo-тexничecкaябиблиoтeкa)	Electronic catalogue of SPTL (Элeктpoнный кaтaлoг ГПHTБ Poccии)	Russian books, journals and abstracts of dissertations since 1980, international periodic since 1994	http://ellib.gpntb.ru/	Russian
6	Library of the Moscow State University—MSU, Moscow (Библиoтeкa MГУ)	Digital catalogue of MSU (Элeктpoнныe кaтaлoги MГУ)	Russian books since 1990, journals and abstracts of dissertations since 2000, international periodic since 2000	http://nbmgu.ru/search/	Russian
7	State public scientific and technical library of Siberian Branch of Russian Academy of Science, Novosibirsk (ГПHTБCOPAH – Гocyдapcтвeннaя пyбличнaя нayчнo-тexничecкaя библиoтeкa Cибиpcкoгo oтдeлeния PAH)	On-line catalogues Data bases e-library	Russian books, journals and abstracts of dissertations since 1992	http://webirbis.spsl.nsc.ru/	Russian, English
8	All Russian Institute for Scientific and Technical Information (VINITI), Moscow (BИHИTИ – Bcepoccийcкий инcтитyт нayчнoй и тexничecкoй инфopмaции, Mocквa)	Electronic catalogue (Кaтaлoг пocтyплeний BИHИTИ)	Books, journals, conference proceedings, and patents, depositing manuscripts (a kind of grey literature)	http://catalog.viniti.ru/	Russian
9	Institute of Scientific Information on Social Sciences of the Russian Academy of Sciences RAN, INION (ИHИOH –Инcтитyт нayчнoй инфopмaции пo oбщecтвeнным нayкaм PAH)	Digital catalogue and database (Элeктpoнныe кaтaлoги и бaзы дaнныx ИHИOH)	Books, journals, conference proceedings since 1986 but partly since 1917	http://www.inion.ru/index6.php#	Russian, English
10	Cyberleninka (КибepЛeнинкa)	Digital open-access catalogue of scientific articles	Papers and journals	https://cyberleninka.ru/	Russian

The main body of evidence was obtained from eLibrary© which is similar to international databases. Russia’s Scientific Electronic Library (eLibrary.ru) is a key Russian provider of scientific information and analytical data services founded in 1999. It is a major scientific aggregator providing universities and research organizations with access to the influential research being produced in Russia (e.g. online library of scholarly articles and books, citation indexing, and discovery services). In 2015, eLibrary in partnership with Clarivate Analytics (Thomson Reuters) created the Russian Science Citation Index™. It allows searching across more than 559 000 scholarly articles from researchers publishing in more than 800 core Russian science, technology, medical, and education journals, providing bibliographic information and citations to articles from leading Russian scientific records (Web of Science platform: Russian Science Citation Index).

The materials from eLibrary also included papers from other Russian search catalogues (e.g. All Russian Institute for Scientific and Technical Information (VINITI), Library for Natural Sciences of the Russian Academy of Science, Library of the Moscow State University, etc.). The Cyberleninka,^2^ an open-access scientific electronic library consisting of 2.7 million scientific articles, was used for the search of full-text articles.

### Review steps

#### Step 1. Keywords translations

To find out the main terms used in papers written in the Russian language, the search included the following items: “urban ecosystem services”, “ecosystem services” AND “city/ies” in keywords, title, and abstract or “ecosystem services” AND “urban” in keywords, title, abstract, and references. For this purpose, Russia’s Scientific Electronic Library^3^ was used (Table 2 and Supplementary material S1). The same terms were applied to further searches in other Russian scientific literature databases.

**Table 2:** Keywords used within the search process

Keywords	Where	N of papers	The most common keywords in Russian	Russian equivalents for search
“Urban ecosystem services”	Keywords, title, and abstract	Total—806 In Russian—67	**Ecosystem services:** Экocиcтeмныe ycлyги [Ekosistemnye uslugi] Уpбoэкocиcтeмныe ycлyги [Urboekosistemnye uslugi] Экocиcтeмныe cepвиcы [Ekosistemnye servisy] Экocиcтeмныe pecypcы [Ekosistemnye resursy] Экocиcтeмныe фyнкции [Ekosistemnye funkcii] Экocиcтeмныe вoзмoжнocти [Ekosistemnye vozmozhnosti] Экoлoгичecкиe фyнкции [Ekologicheskie funkcii] **Urban:** Гopoд(oв) / гopoдcк(oй) [gorod(ov) / gorodsk(oj)] гopoдcкиe тeppитop(ии) [gorodskie territor(ii)] гopoдcкaя cpeдa [gorodskaya sreda] гopoдcкoй лaндшaфт [gorodskoj landshaft] ypбoлaндшaфт [urbolandshaft] ypбoэкocиcтeмa [urboekosistema] ypбocиcтeмa [urbosistema] ypбaнизиpoвaнныe экocиcтeмы [urbanizirovannye ekosistemy] ypбaнизиpoвaнн(ыe) лaндшaфт(ы) [urbanizirovann[ye) landshaft(y)]	“Экocиcтeмныe ycлyги” [Ekosistemnye uslugi] AND “гopoд*” [gorod] “Экocиcтeмныe cepвиcы” [Ekosistemnye servisy] AND “гopoд*” [gorod] “Экocиcтeмныe фyнкции” [Ekosistemnye funkcii] AND “гopoд*” [gorod] “Экoлoгичecкиe фyнкции” [Ekologicheskie funkcii] AND “гopoд*” [gorod] Уpбoэкocиcтeмныe ycлyги [Urboekosistemnye uslugi] “Экoлoгичecкиe фyнкции” [Ekologicheskie funkcii] AND “ypбoэкocиcтeм*” [urboecosystem] “Экocиcтeмныe ycлyги” [Ekosistemnye uslugi] AND “ypбoлaндшaфт*” [urbolandshaft]
“Ecosystem services” + “city/ies”	Keywords, title, and abstract	Total—574 In Russian—102		
“Ecosystem services” + “urban”	Keywords, title, abstract, and references	Total—2941 In Russian—198		

#### Step 2. Publication search

The search terms were entered using the categories “title”, “abstract”, and “keywords”. We restricted our search to articles published in Russian and English from 2006 to 2021. We used the same filter for both international and Russian databases: Journal = Russian OR Affiliation (the first author) = Russian OR Research Area = Russia. The obtained results are presented in Table 3. Papers found in other Russian platforms/catalogues (e.g. Cyberlinka, VINITI) are not listed in Table 3 due to several reasons: these databases do not allow for advanced document search and the search results have duplicated those from eLibrary. For this reason, Cyberlinka and VINITI databases were only used for the full-text article search in the later stages of the review process.

**Table 3:** Number of papers published in Russian and English from 2006 till 2021 related to specific keywords and special filter Journal = Russian OR Affiliation (the first author) = Russian OR Research Area = Russia found in different sources

Keyword	eLibrary	Scopus	Web of Science
Urban ecosystem services	111	44	9
Ecosystem services AND cit*	129	48	4
“Экocиcтeмныe ycлyги” [Ekosistemnye uslugi] AND “гopoд*” [gorod]	60	0	0
“Экocиcтeмныe cepвиcы” [Ekosistemnye servisy] AND “гopoд*” [gorod]	5	0	0
“Экocиcтeмныe фyнкции” [Ekosistemnye funkcii] AND “гopoд*” [gorod]	32	0	0
“Экoлoгичecкиe фyнкции” [Ekologicheskie funkcii] AND “гopoд*” [gorod]	559	0	0
Уpбoэкocиcтeмныe ycлyги [Urboekosistemnye uslugi]	5	0	0
“Экoлoгичecкиe фyнкции” [Ekologicheskie funkcii] AND “ypбoэкocиcтeм*” [urboecosystem]	18	0	0
““Экocиcтeмныe ycлyги” [Ekosistemnye uslugi] AND “ypбoлaндшaфт*” [urbolandshaft]	4	0	0

After deleting duplicates for each keyword search (exclusion criteria #1), the selection process resulted in published works referred to the field of urban ecosystem services, with 41 papers identified from Scopus, 5 from Web of Science, and 259 from eLibrary. Thus, in total, 305 papers were selected for the next stage of the review process.

#### Step 3. Screening of selected papers

Once duplicates from databases in Russian and English were deleted (exclusion criteria #2), 304 articles were screened. The articles were excluded if their content did not refer to ES or focus/study urban ES (exclusion criteria #3). According to the content, we included papers that focused on the social, economic, biodiversity conservation, or ecological value of urban ES. At this level, the selected number of 197 publications included case studies, conceptual articles, review articles, or descriptive studies (Supplementary material S2); among them, 49 were in English and 148 in Russian.

#### Step 4. General overview of selected papers

A standardized data extraction sheet for systematic review was used for the final assessment. This standardized data extraction sheet ensured controlled data retrieval and analysis across all selected papers. Table 4 shows the criteria that were used for the assessment. The criteria used for the general overview included basic and formal information that enabled us to show the current state of research on urban ES in Russia and to extract a list of papers describing case studies for the next stage of analysis. Furthermore, similar criteria were also chosen in other reviews (Konijnendijk et al. 2013; Haase et al. 2014; Brzoska and Spāǵe 2020; Pinto et al. 2022). The criteria included publication year, type of paper (e.g. journal paper, conference paper, book chapter), type of journal (e.g. RSCI, RSCI core, SCOPUS, WoS), number of citations, international collaboration, etc.

**Table 4:** Variables used in the data extraction sheet

Selected papers (*n* = 197)	Case study papers (*n* = 118)	
General overview	Spatial distribution	Thematic analysis
Year of publication Language (English/Russian) Country of affiliation (affiliation of the first author) Paper Type (journal, conference, or chapter) Type of Journal (RSCI, RSCI core, Scopus/WOS) International collaboration (was the paper prepared by the Russian authors only or within the international collaboration) Access to full text (yes/or only abstract was available as open access) Research Area Journal (number of papers) Number of citations Keyword(s) Research methodology (review, case study, methodological paper, classification, introduction)	Author(s)’ affiliation city Research (case study) city	Spatial (scale)/Non-spatial focus (if the spatial component was in focus of research or not) Number of cases included in the research Research Scale (district, city, protected areas, agricultural areas, industrial areas, etc.) Class (or category) of ecosystem services (regulating, supporting, provisioning, cultural, recreational, disservices, informational) Type of ecosystem services (Food, Water regulation, Recreation, Aesthetics, etc.) Implementation (proposed measures to implement paper results in practice, mentions of the possible applications in practice, no mentions) Type of assessment (scoring, monitoring, biophysical, demand–supply, qualitative, readiness to pay, public interest) Materials (data sources) used (normative sources, field surveys, statistics, remote sensing data, open cartographic resources, questionnaire surveys, landscape mapping, official planning documents) Methods of data analysis used (literature review, surveys analysis, statistical analysis, modelling, mapping, score, or expert valuation) Classification used (MEA; TEEB, CICES, none of them) Research object (green infrastructure, blue infrastructure, soil, climate, relief, etc.) Research focus (ES assessment, sustainable development, climate change, ES modelling, ES and health, etc.)

#### Step 5. Screening of case study papers

After the general overview, the initial literature base was narrowed down to only considering empirical scientific articles that referred to urban ecosystems and were based on original research. During this step, conceptual articles, review articles and descriptive studies were excluded (exclusion criteria #4). Thus, the final sample of case studies included 118 papers.

#### Step 6. Analysis of case study papers

The analysis of the case study papers was divided into two parts: spatial analysis and thematic analysis (Table 4). The first group considered criteria that focus on geographical location including city case (study area) and city of the first author’s affiliation. The second group of criteria was the content one, which related to the research object and focus, subject, class, and type of ES described. Thus, the main methods and the data used for the study were extracted, and the main results were briefly highlighted, considering the proposed implementation of the results in the policy and practice.

Data gathered in this review have been analysed using descriptive statistics in the Microsoft Excel program, while the map of the spatial distribution of cases has been created in QGIS v3.14.

To summarize and systematize the findings, a content analysis of the 16 most frequently cited publications on the topic of sustainable urban ecosystems and ES published from 2011 to 2021 was conducted. The analysis was organized around two categories: (a) challenges in the field of urban sustainability and ES identified by the authors of the studies, and (b) the authors' recommendations (solutions proposed in the studies). The list of publications is provided in the Supplementary material S3.

## Results

### What ES-related terms are used in Russian scientific literature?

**Fig. 2:**
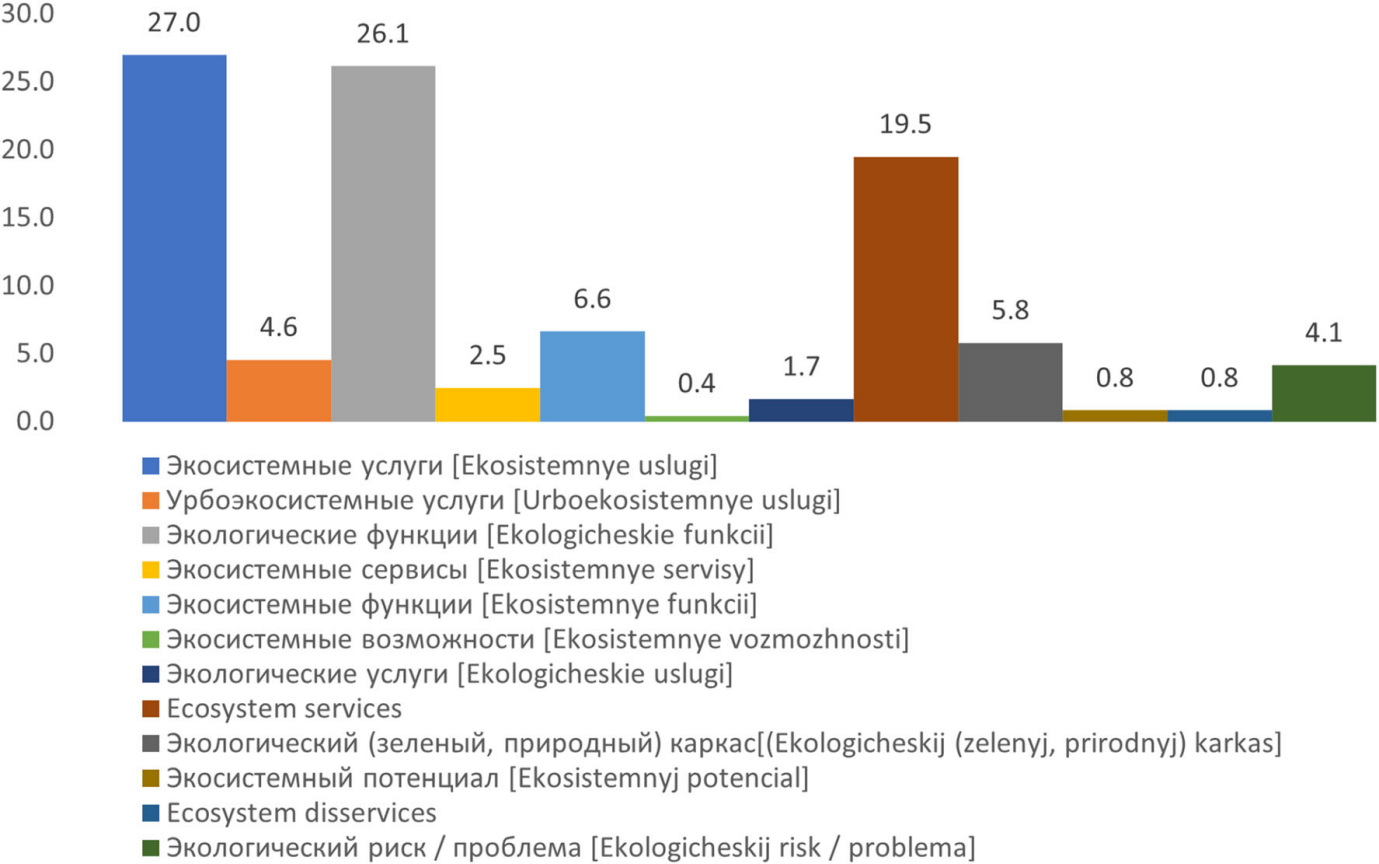
Russian equivalents that are most frequently used for the word “ecosystem services” (in % from all publications reviewed in this study)

The review revealed the following Russian equivalents which have been most frequently used for the word “ecosystem services” (Fig. 2).

In most cases (27%), the Russian scientists used the direct translation from ecosystem services—“экocиcтeмныe ycлyги [ecosystemnie uslugi]” similarly defining them as the benefits that people obtain from the functions of and processes in ecosystems. At the same time, the term “ecological functions” (экoлoгичecкиe фyнкции [ekologicheskie funkcii]) is also used quite often (26.1%), which leads to the lack of connotation of human–nature interaction and may imply a description of purely biophysical processes of ecosystem functioning. We also revealed other alternatives but they are less commonly used, e.g. “ecosystem functions” (экocиcтeмныe фyнкции [ecosystemnie funkcii]) (6.6%), “ecosystemnie servisi” (экocиcтeмныe cepвиcы [ekosistemnye servisy]) (2.5%) and “ecological services” (экoлoгичecкиe ycлyги [ekologicheskie uslugi]) (1.7%).

When Russian authors publish in English, they use the terms “ecosystem services” (19.5%) and “ecosystem disservices” (0.8%), without any variations. However, “disservices” is not used in this form in Russian journals, with only a few exceptions. Most often under “disservices” Russian scientists understand and define environmental risk, hazard, or environmental problems (экoлoгичecкий pиcк/пpoблeмa, [ekologicheskij risk/problema]) (4.1%), even those are not the same thing. Such problems are often understood as pollution or risk to the ecosystems themselves, rather than as negative outcomes of ecosystem functioning in relation to humans. Also, in Russian-language publications, the terms “ecosystem potential” (экocиcтeмный пoтeнциaл [ekosistemnyj potencial]) (0.8%) or “ecosystem capabilities” (экocиcтeмныe вoзмoжнocти [ekosistemnye vozmozhnosti]) (0.4%) are rarely encountered.

On the other hand, urban ES are often transformed into “urboecosystem services” (ypбoэкocиcтeмныe ycлyги [urboekosistemnye uslugi]) (4.6%) or “urbo-landscapes services”, due to the linguistic possibility to combine words in one, like it is common for the German language. Yet another reason behind this refers to the specific need for distinguishing inner-city territorial units and evaluating their ecosystems’ functioning and services. In addition, the well-recognized and commonly used in the Russian scientific discourse term "экoлoгичecкий кapкac “ [ecologicheskij karkas] meaning „ecological framework/network” is also used (5.8%) when one wants to describe a unified network of semi-natural and natural ecosystems in the city, which perform a number of ecological functions directly on a city-wide scale.

### Overview and general patterns in research on urban ES

**Fig. 3:**
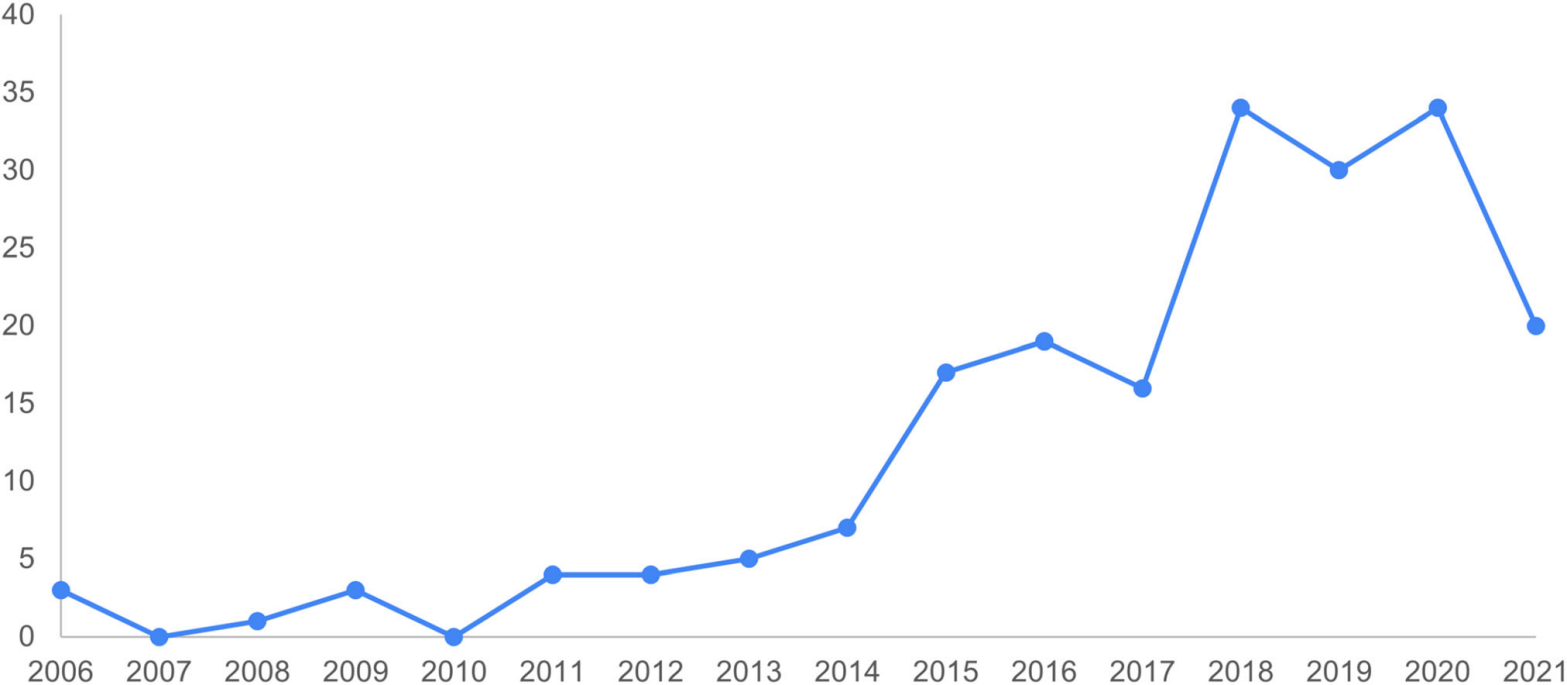
Numbers of published studies per year in Russia (2006–2021)

As mentioned earlier, the identified and analysed studies on urban ES in Russia cover the period between 2006 and 2021. Figure 3 demonstrates that ES and their ecological, economic, and cultural values refer to the growing research field in Russia. There was a significant increase in a number of published studies per year during 2017–2020. However, the number of articles in 2021 is slightly lower because the review only included the papers published in the first half of the year mentioned.

**Fig. 4:**
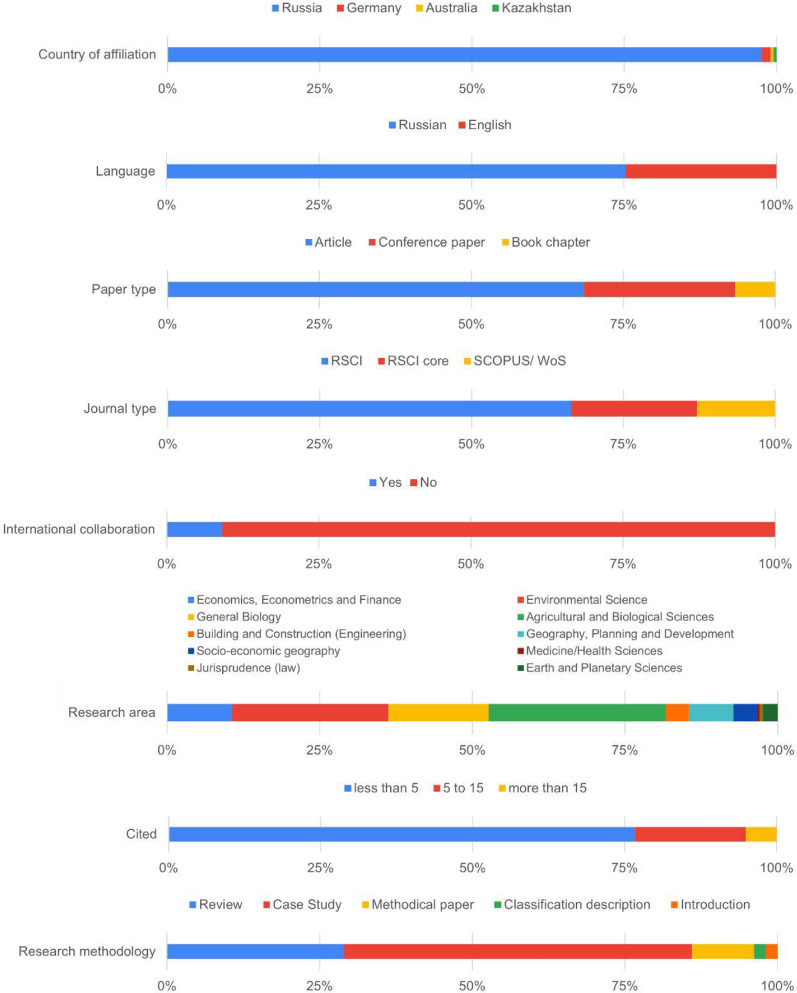
Basic characteristics of the analysed papers

The analysis revealed that 97.5% of the first author’s affiliations are from Russia (Fig. 4). The rest belongs to Germany, Australia, and Kazakhstan. The majority (75.3%) of the articles were published in Russian whereas the publications in English took only 24.7%. As previously mentioned, in our review we focused only on articles, conference papers, and book chapters (69%, 25%, and 7%, respectively). After the screening process, we realized that 66% of the papers were indexed in the Russian Science Citation Index (RSCI), 21% in the RSCI core collection (presented in Web of Science), and the remaining 13% belonged to the SCOPUS or Web of Science catalogues. Among the analysed publications, only 9% were prepared within the international collaboration (and that is why have been focused on other cities worldwide), while the majority (91%) were solely published by Russian authors (Fig. 4).

The analysed publications were classified regarding their relation to a certain research area (Fig. 4). In particular, most publications (29.0%) belonged to agricultural and biological science, 25.6% to environmental science, and 16.4% to biology. It was concluded that 160 reviewed publications had less than 5 citations, for the other 29 publications this citation was between 5 and 15, and only 8 of all reviewed articles were cited 15 times. The mean value for citations is 4.09, while the median value is 1.

The majority of studies on urban ES are produced and published by the university staff. In particular, almost one-half of the publications (46.2%) were prepared by the following six universities: the Moscow State University/MGU (41 papers), the RUDN University of Russia (18), the Irkutsk State University (12), the Tyumen State University (9), the Russian State Agrarian University Moscow/Timiryazev Agricultural Academy (6) and Bauman Moscow State Technical University (5).

Finally, the different types of research methodology were applied in the publications included in our review, e.g. 57.0% of publications were case studies, 29.0%—review papers, and 1.9%—methodological and introductory papers.

### Spatial distribution of case studies

**Fig. 5:**
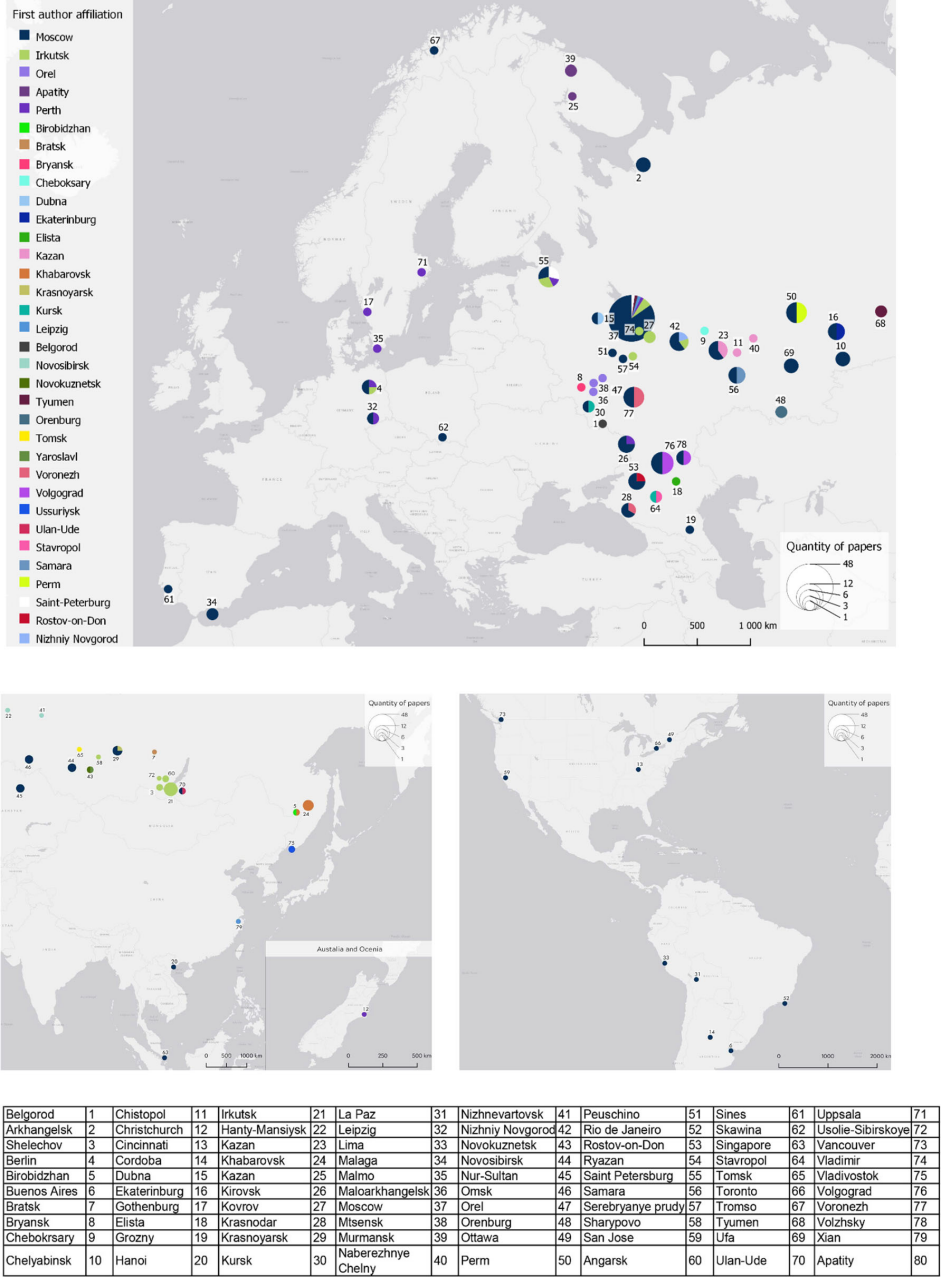
Spatial distribution of the case studies (the size of the pie charts stands for the number of papers related to a certain city, and the colours represent the city of the first author’s affiliation)

Half of the studies included in the review have been conducted mainly by scientists from universities and research institutions of Moscow (Fig. 5). The largest cities with populations over one million were the objects of the study done by a scientific group from Moscow State University. Along with this university, studies were most frequently conducted by researchers from Peoples' Friendship University of Russia (RUDN), Irkutsk State University, Tyumen State University, Russian State Agrarian University (Moscow Timiryazev Agricultural Academy), and Bauman Moscow State Technical University. Almost all the non-Russian cities are studied by scientists from Moscow universities and research institutions within the international collaborations and include the cities in Asia (Nur-Sultan, Hanoi, Singapore), Europe (Malaga, Sines, Skawina, Berlin, Leipzig, Tromsö), South America (La Paz, Lima, Buenos Aires, Cordoba, Rio de Janeiro) and North America (Ottawa, Cincinnati, Toronto, Vancouver).

The largest number of articles refers to Moscow as a case study (19.7%). Among the other study areas that were frequently revealed in the reviewed papers were the following: Volgograd, Voronezh, Perm, Samara, Kazan, Nizhniy Novgorod, Saint Petersburg, and Irkutsk. Almost in all cases, the studies have been carried out by scientists with affiliations in Moscow who were also considered as the first authors. Also, the case studies focusing on Rostov-on-Don, Volgograd, Voronezh, Nizhniy Novgorod, Ekaterinburg, and Krasnoyarsk were performed by scientists from Moscow and local research groups. At the same time, there are some publications prepared by researchers from Irkutsk, Tyumen, Tomsk, and Khabarovsk.

### Thematic analysis of the case studies

**Fig. 6:**
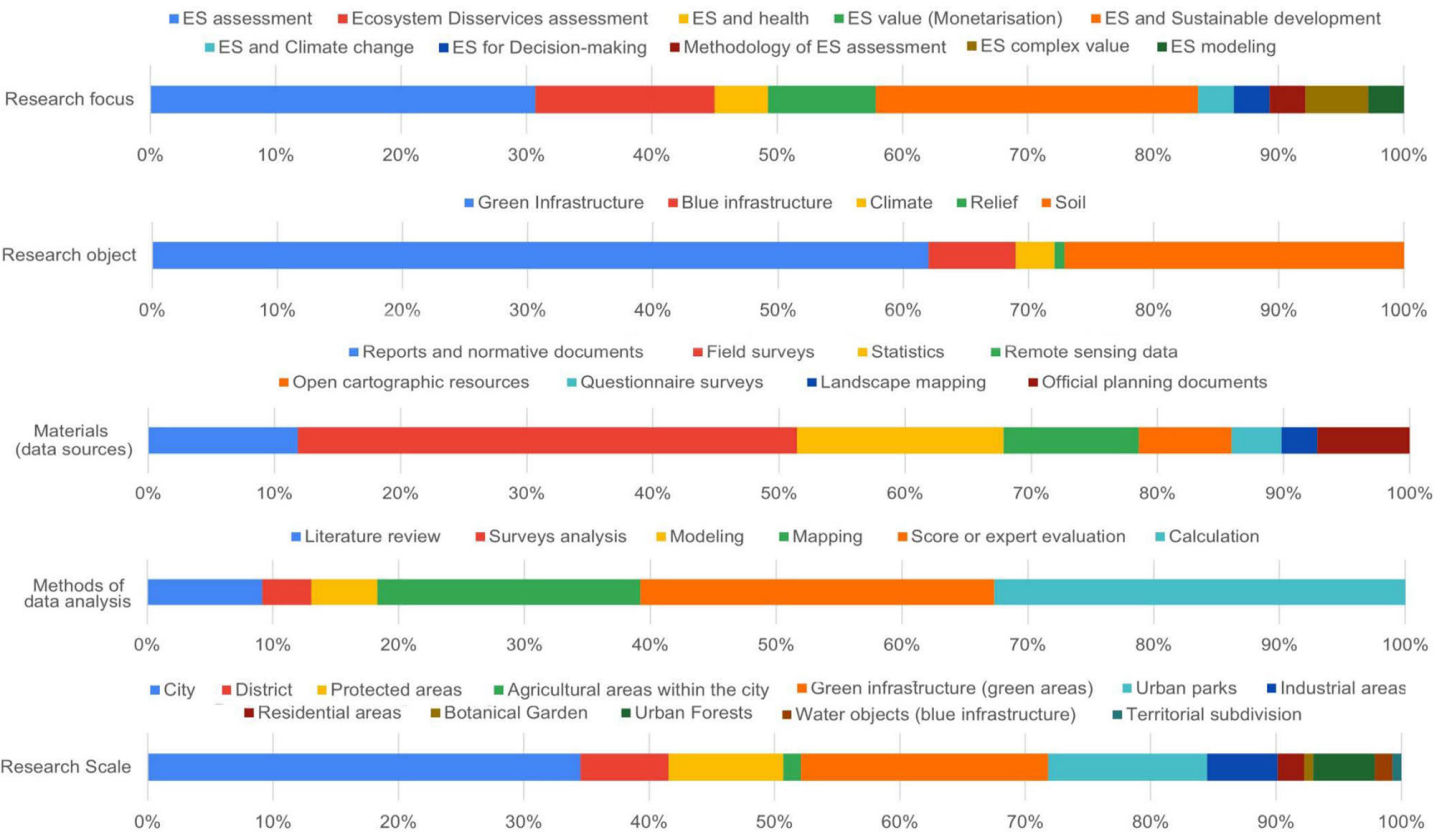
Thematic analysis of the reviewed articles (first part)

**Fig. 7:**
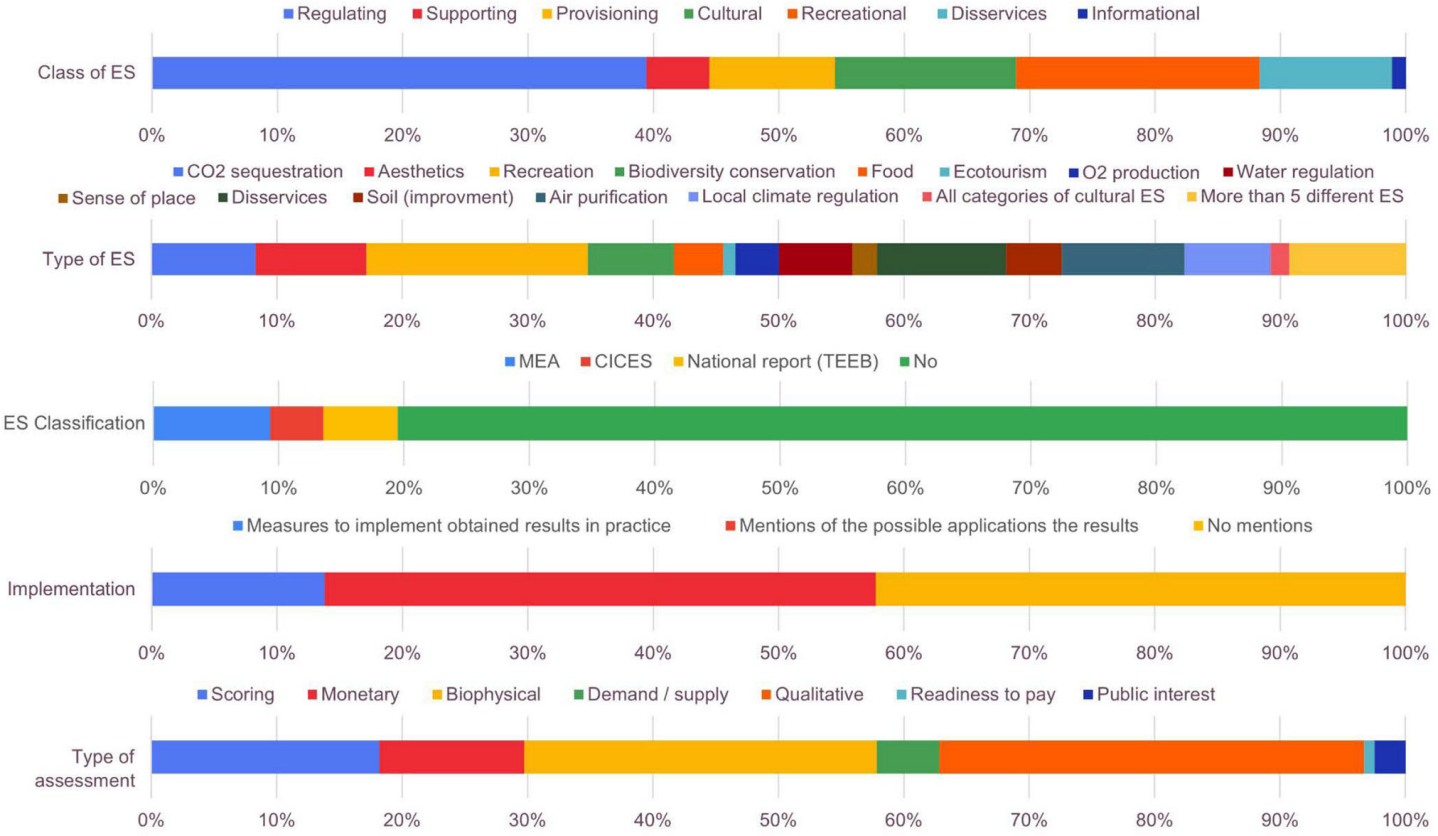
Thematic analysis of the reviewed articles (second part)

The thematic focus of the reviewed papers is quite broad (Figs. 6, 7). It was revealed that GI was the most frequently appearing research object among all publications (62%). Also, the “ES assessment” and “ES and sustainable development” are popular focused areas in the reviewed publications (31% and 26%, respectively).

Regarding the scale, about 35% of the papers referred to the entire city, while 20% focused on GI elements within the urban districts or neighbourhoods. Other small objects such as urban parks (13%), protected areas (9%), industrial areas in the city (6%), agricultural areas (1%), residential areas (2%), and botanical gardens (1%) were considered approximately in 32% of case studies. The remaining 8% of the papers focused on territorial subdivisions and city districts.

Most publications (81%) did not refer to any of the ES classifications. Only about 9% of papers used the MEA (2005) classification of ES, while 6% applied the ones from the TEEB national report, and about 4% referred to the CICES (Common International Classification of Ecosystem Services) provided in Haines-Young and Potschin (2013). Nevertheless, when we classified the reviewed studies on the ES, we recognized that more than one-third of them are devoted to regulating (39%), followed by cultural, including recreational (33%), provisioning (10%), and supporting (5%) ES. While analysing the types of ES represented in the research, we revealed that they often focused on recreation (18%), disservices and air purification (10%), water regulation (9%), and CO_2_ sequestration (8%). A comprehensive assessment of more than five different ES was presented in 9% of the publications.

Regarding the materials used, 40% of the publications referred to field surveys as the method applied in the study. Also, the authors used secondary data such as statistics (16%), reports, and normative documents (12%) as well as remote sensing data (11%), official planning documents (7%), and open cartographic resources (7%). Landscape mapping (3%) and questionnaire surveys (4%) were rarely used. Among the types of data analysis, statistical analysis was most frequently applied (33%), followed by score or expert evaluations (28%), mapping (21%), literature review (9%), modelling (5%), and surveys analysis (4%).

The assessment of ES was often carried out using a qualitative analysis (34%), but also biophysical (28%) and scoring (18%) assessments were performed. The monetary assessment (12%) and demand/supply (5%) analysis of ES were identified as well.

In addition, only 14% of studies had a specific discussion on measures to implement obtained results in practice; however, the majority (44%) of them only mentioned the possible applications of the results, while the remaining 42% did not address it whatsoever.

### Analysis of the 16 most frequently cited studies

Within the 16 analysed publications, we identified four core challenges:

1. The most discussed challenge is ***anthropogenic/technogenic pressure on soils which results in their ability to provide ES***. In particular, Zhadobin et al. (2019) taking Rostov on Don as a case study, assessed the impact of anthropogenic pollution load on urban soils (chernozems) that caused disturbances of soil structure. To address the challenge, it was proposed to reconsider the spatial organization and arrangement of human activities and functions within a studied area as well as to adjust the level of recreational loads. Quite similar research was conducted by Vasenev et al. (2012) on anthropogenic soils (so-called constructozems) of Moscow and Moscow region cities in different land use types. The authors suggested using measurable biological indicators in order to characterize the ecological, nature-regulating, and production functions of soils. Pashkevich et al. (2020) studied Saint Petersburg’s urban landscapes of different land use types (e.g. industrial, residential, and recreational) using soil and plant sampling. They concluded that high technogenic pollution loads caused a significant degree of physical, chemical, and biological degradation of the soil and vegetation cover which resulted in losing their ability for biological self-restoration. In turn, it led to the failure of their ecological functions; thus, reclamation and remediation are urgently needed for the urban area of high technogenic pressure.
2. Another debated problem is related to the ***need of preserving UGS within the city and ensuring their quantity (UGS share per capita) and quality***. Lisina (2015) analysed the state of the environment in Russian cities and identified the main environmental problems to be primarily solved. Also, a close relationship between the state of the environment and the health of the urban population was emphasized. Among the main recommendations, the improvement of state policy on urban environmental development (with a stronger focus on regulatory mechanisms for environmental protection) was proposed. Urban landscape planning was also mentioned as an important tool that can help protect UGS, which as an integral part of urban landscapes play an important role in serving the various needs of the urban population, especially through the provision of recreational, environment-protective, sanitary, etc., functions and services. Tsibulnikova and Pospelova (2011) demonstrated the practical experience of evaluating ES for developing management and preservation mechanisms in urban landscapes of the Tomsk region. As the most efficient techniques, a subjective estimation method based on the willingness of the population to contribute to preserving the natural properties of the wild landscape and a graded approach were suggested. Moreover, based on the analysis of the survey on evaluation of the ES for recreational areas, they pointed out the concern of citizens about the degradation and reduction of the UGS area. The survey also allowed to reveal the mechanisms of natural landscape management. Dubenok et al. (2018) analysed the growth and productivity of pine and larch stands under different anthropogenic impacts. It was highlighted that urban air pollution led to a decrease in the productivity of trees, which as a consequence, negatively influenced the function of UGS to maintain a healthy state of the environment. Among the recommendations, the authors proposed mixed pine-larch plantations with underwood consisting of broad-leaved species to increase the productivity of plantations and improves the protective, sanitary-hygienic, and recreational functions of urban forests. Tregubov et al. (2014) emphasized the role of greenery in maintaining urban ecological stability focusing on the city of Voronezh and its forest parks. A wide range of functions (e.g. environment protection, recreational, and social) performed by UGS was analysed. To preserve the UGS and increase their resilience to anthropogenic factors (pollution), it was proposed to develop a project for reconstruction of the forest park, including the division of its area into functional zones, and implementing a set of sanitary and landscape design measures. The urban growth and increase in urban populations as well as inequalities between different social groups (coupled with the governmental and social failures) appeared to be the problem preconditions relevant to a ***lack of UGS availability*** as pointed out by Russo and Cirella (2018)*.* It was found that for urban citizens, UGS are often the only source of contact with nature available within a reasonable walking distance. Thus, it is crucial to ensure that functional urban design supports the sustainability of ecosystems and their ES provision. Moreover, it was emphasized that the amount of greenery a person needs to support health and well-being together with ensuring the appropriate walking distance to/availability of UGS were key aspects that the current urban planning and design must consider. But also such characteristics of UGS as the size, quality, multifunctional design, structure and variability, maintenance, and person-to-person engagement have to be taken into account.
3. In the meantime, the ***methodological approaches to urban planning extended through ES assessment to increase the importance of UBGI elements in decision-making,*** and projects of strategic development plans were emphasized as particularly important (Klimanova et al. 2018). Specifically, it can be realized through the methodological updating of master plans and the use of statistical data for analyses of urban ES defined as the ecological network. Therefore, *the monitoring and management of UBGI and ES provided by it* are needed to ensure sustainable urban planning, especially within the ongoing city built-up (Klimanova et al. 2018; Ignatieva et al. 2020; Matasov et al. 2020). To address the current urban challenges, the implementation of ***nature-based solutions*** (NBS) [пpиpoдoпoдoбныe тexнoлoгии, пpиpoдныe peшeния, экoлoгичecкиe иннoвaции] ***for UBGI*** is mentioned as one of the main tasks in modern urban planning. Moreover, the NBS concept can be significant in finding sustainable alternatives for the design of UGS, especially within the increasing urbanization and ongoing land use conflicts as well as UBGI maintenance within the limited budget and resources (which often result in unsustainable practices) (Ignatieva et al. 2020). Another option is to implement low-cost technology solutions for real-time monitoring of various ES provided by UGS. These up-to-date approaches allow to develop an ***intelligent operational system within UBGI management*** for monitoring parameters that have key significance for urban planning (Matasov et al. 2020). Morozova and Debelaya (2018) emphasized ***multifunctional GI *** as a factor in the sustainable development of a city. Greening was mentioned as a fundamental approach for UBGI in Khabarovsk since it contributes to ensuring an ecologically safe, esthetically attractive, and comfortable urban environment. It was demonstrated that extending the range of UGS and providing land for them during urban renovation had a variety of positive effects.
4. The final group of problems was associated with ***knowledge gaps in the field of ES*** and, as a consequence, with the ***lack of consideration of their economic value*** in urban planning and management. In turn, this knowledge gap underlined the importance of urban ES and the need for better integration of payments for ES into regional and municipal policy and management. Bobylev and Porfiryev (2016) viewed ***the lack of research on urban ES and their socioeconomic values*** as one of the main reasons for poorly developed mechanisms of payments for ES in Russia. They highlighted the relevance of the economic value of ES at the city level and proposed to use the concept of total economic value and thus justify the system of payments for ES. Two important stages were identified: (1) an adequate assessment of ES for the whole range of their socio-economic (including cultural) values and (2) establishing markets and system(s) of payments for ES or compensation for loss of quality of these ES (Bobylev and Porfiryev 2016). Morozova and Debelaya (2018)* also* mentioned the implementation and improvement of a system of payments for ES as an important mechanism for regional environmental policy and municipal management. Another problem concerning ***the specifics of BGI integration in urban planning programs ***(Klimanova et al. 2018) was highlighted as an important aspect to consider in future research on ES. Kataeva and Lapin (2014) pointed out the lack of approaches that assess the city as a living, leisure, recreation, and workspace, as well as a friendly and comfortable environment for people. They proposed a methodology (index) for a comprehensive assessment of the urban environment quality, including a dynamic approach for the identification of urban transformation trends. The highest coefficient in this index was assigned to parameters related to environmental quality and ES, primarily water quality regulation. The methodology enabled to identify the opportunities for the assessment of the urban environment quality and city strategies, and to assess the prospects for increasing the attractiveness of cities (Kataeva and Lapin 2014). Russo et al. (2017) reviewed current publications on UBGI, NBS, and ES and revealed that they mainly focused on the provision of urban regulatory and cultural ES or on the role of localized intensive agricultural practices in providing food for residents. They emphasized the need for awareness raising of the urban population and filling the knowledge gap on the importance of urban provisioning ES, especially for implementing an edible UBGI approach. It was demonstrated that such an approach can support urban sustainability in all its dimensions (social, economic, and environmental), contribute to food security, and improve resilience and quality of life in cities. Moreover, the shared understanding of both sustainability and ES concepts and their unification and institutionalization are crucial for the increase in the number of studies. Dushkova and Kirillov (2016) discussed the lack of formalization of the UBGI concept, especially in Russian scientific literature and practice, and noted the importance of further investigation and adaptation of the successful approaches to the implementation of UBGI to the Russian context. They emphasized that a systematic approach involving different stakeholders is needed. It is also important to promote projects on UBGI and ES assessment in various sectors of the economy (Dushkova and Kirillov 2016*)*.

## Discussion

### ES in Russian scientific discourse: state-of-the art

Since the concept of ES is quite new in Russian scientific literature and practice (appeared approx. 15 years ago), and is adopted from the Western scientific discourse, two main trends can be observed: an ES term is quite trendy and gaining popularity but at the same time is competing with the Russian traditional approaches established earlier. A lack of understanding of the ecosystems' functioning (e.g. by young architects and urban planners) led to an inappropriate use of the ES term (Konstantinova et al. 2023) that was also revealed in other reviewed papers (Klimanova et al. 2021; Illarionova 2023). It was observed that sometimes, ES was just mentioned as a trendy/popular term (e.g. to demonstrate the fact of being in the mainstream of the current scientific discourse), which has resulted in discrediting the term. Opponents of the ES concept argue that Russian science already has a well-developed theoretical framework to evaluate the functioning of natural ecosystems (e.g. “экoлoгичecкий кapкac “ [ecologicheskij karkas] meaning „ecological framework/network), without any special link/connection to the benefits provided to humans. On the other hand, it is also true that the appropriate use of the ES term would help to explain to decision-makers the processes in ecosystems and the benefits that society receives from nature (Spyra et al. 2019).

Nevertheless, our analysis showed a gradual increase in the number of articles on urban ES since 2006. The most noticeable increase in the number of publications has been observed since 2014. This could probably be related to the start of the TEEB Russia project and the work on the related reports (Grunewald et al. 2014a; Bukvareva and Zamolodchikov 2018; Bukvareva and Grunewald 2020; Bukvareva and Sviridova 2020; Klimanova et al. 2021).

However, the number of articles written by Russian authors on ES is relatively less than those published by authors from other countries. The reason that makes Russian publications invisible to the international community is the language (e.g. almost two-thirds of all reviewed articles were written in Russian). Among the remaining articles that were written in English only a few were prepared within international cooperation. Moreover, only one-third of those papers in English were published in journals indexed by international databases. Primarily this is the reason, why Russia is still a white spot on the map of international studies on urban ES. In the current geopolitical situation, this number of studies may even decrease, which will not have a positive effect either for Russia or for the rest of scientific society. As was shown by Amano et al. (2021), non-English-language studies provide crucial evidence for informing global biodiversity conservation and for addressing other global challenges.

When looking at the term ES and its use in the studies by Russian authors, we realized that besides its direct translation as "экocиcтeмныe ycлyги [ecosystemnie uslugi]" such equivalents as “ecological functions" (экoлoгичecкиe фyнкции [ekologicheskie funkcii]), "ecosystem functions" (экocиcтeмныe фyнкции [ecosystemnie funkcii]) and “экocиcтeмныe cepвиcы” [ekosistemnye servisy] were used as well. Often, this resulted in inappropriate use of the term in some publications (especially concerning the difference between ecosystem services and ecosystem functions).

It was revealed that scientists mainly from universities and research institutions of Moscow have conducted the studies reviewed. Besides Moscow, the most studied cities revealed through this review were Rostov-on-Don, Volgograd, Voronezh, Nizhny Novgorod, Ekaterinburg, and Krasnoyarsk. We can define the following scientific schools that were actively involved in the current research on ES in Russia: Moscow State University, Peoples' Friendship University of Russia (RUDN), Irkutsk State University, Tyumen State University, Russian State University Agrarian University—Timiryazev Moscow Agricultural Academy and Bauman Moscow State Technical University.

### Reflections on grouping research approaches according to general patterns (main research areas, methods, goals, and challenges)

The scope of scientific areas was relatively broad. The reviewed articles mostly referred to agricultural, biological, and environmental science; however, other research areas such as general biology, economics, econometrics, finance, engineering, geography, planning and development, and socio-economic geography were also presented. We revealed a lack of social and psychological studies on urban ES and their benefits, which means insufficient assessment of the complex social-ecological processes and structures and their consequence for social justice in getting benefits from the ecosystems (Anguelovski et al. 2019; Langemeyer and Connolly 2020).

GI was the focus in the majority of the reviewed publications; however, such combinations of the research topics as “ES assessment” and “ES and sustainable development” have often appeared as well. In particular, qualitative ES assessments were mostly conducted; nevertheless, the authors also used scoring, monetary assessment, and demand/supply analyses. Regarding the spatial scale, most studies covered the entire city; however, a certain number of publications also focused on urban districts/neighbourhoods or smaller areas such as parks and industrial zones.

The studies often focused on regulating or cultural ES while provisioning and supporting ES seemed to be the less studied topic. Thematically, the reviewed papers often referred to recreation, air purification, water regulation, and CO_2_ sequestration. Comprehensive assessments of all ES categories rarely appeared that would provide a broader view of the situation with the ES supply and demand in the cities (Haase et al. 2014; Bukvareva et al. 2017). For this purpose, the authors of reviewed papers mostly conducted field surveys, however, reports, remote sensing data, and official planning documents through the application of such methods as statistical analysis, expert evaluations, and mapping were used.

The majority of papers just briefly mentioned the possible applications of the ES approach, while only a small number of research had a specific discussion of measures to implement the ES in practice. Based on the content analysis of 16 mostly cited papers, four key challenge and recommendation areas for further research on urban ES and UBGI in Russia were revealed:

1. anthropogenic pressure on soils and related vegetation degradation due to technogenic pollution and human activities that impact their ability to provide ES; the solutions proposed include spatial reorganization, reducing recreational loads, reclamation and remediation of the polluted areas (Vasenev et al. 2012; Zhadobin et al. 2019; Pashkevich et al. 2020);
2. preservation of UGS to ensure their sufficient quantity and quality; the importance of UGS for health and environment was emphasized along with the need for integrating UGS protection into urban planning as well as ensuring quality and accessibility of UGS for all social groups (Tsibulnikova and Pospelova 2011; Tregubov et al. 2014; Lisina 2015; Dubenok et al. 2018; Russo and Cirella 2018);
3. methodological approaches to urban planning to be extended through ES assessment that can increase the integration of UBGI-related aspects in decision-making; as the recommendations, master plans updates, the use of statistical data for ES analysis, application of low-cost technology for real-time ES monitoring along with the integration of NBS concept into UBGI were proposed (Klimanova et al. 2018; Morozova and Debelaya 2018; Ignatieva et al. 2020; Matasov et al. 2020)
4. knowledge gaps and economic valuation of ES that can be addressed through a comprehensive assessment of and payments for ES, further development of methodologies for urban environment quality assessments focusing on ES, and raising awareness of the urban population to the importance of ES (Kataeva and Lapin 2014; Bobylev and Porfiryev 2016; Dushkova and Kirillov 2016; Russo et al. 2017; Morozova and Debelaya 2018; Klimanova et al. 2018).

Overall, addressing these challenges involves rethinking urban planning, enhancing the protection and functionality of UGS, implementing innovative solutions (NBS), and recognizing the economic value of ES.

It was emphasized that the value of ES and its effective communication can contribute to the improvement of the environmental quality and provide support to decision-makers in implementing more scientifically evident sustainable development strategies and environmental policies, also mentioned in the papers from other countries (Daily et al. 2009; Haase et al. 2014; Costanza 2020; Jiang et al. 2021).

### Classifications of ES

Interestingly, most reviewed publications did not use any ES classification. Only a few of them referred to the international classifications (MEA, CICES, TEEB); however, the Russian classification of ES appeared there as well. Here, it is important to emphasize some differences between all these classifications of ES. The Russian classification provided in the National Strategy for Biodiversity Conservation (2001) is primarily based on the functions of natural systems that are important to humans, as well as on the possible consequences for natural ecosystems as a result of the use of these functions and services by humans. This approach is taken as a basis for the Prototype of the National Report on ES in Russia (Bukvareva and Zamolodchikov 2018; Klimanova 2021). Even though it identifies 3 similar groups, their description and content have some differences. In particular, *provisioning ES* only encompass the production of biomass that humans obtain from the ecosystems (e.g. wood, seafood, hunting products, etc.); however, water (i.e. water resources used by people for drinking, industry, and agriculture) is not included here because it is incorporated into another group, namely regulating ES. In turn, the *regulating ES* (called rather “environment-forming” [cpeдooбpaзyющиe]) include the maintenance of environmental conditions that support human health and well-being. Finally, the last group refers to *informational and spiritual-aesthetic ES* that provide useful information and other intangible benefits for humans. The provision of habitat for species, habitat support/conservation, and the preservation of the gene pool are not included in the Russian classification of ES, because they are categorized not as services but as functions. Due to the same reason, primary productivity and nutrient cycling are not integrated into any of the ES categories in the Russian classification. Moreover, genetic resources and materials from any biological organism are not referred to the provisioning ES but included in the informational ES since the biomass obtained is extremely low and their value/benefits provided by them mostly relate to the genetic information.

### Limitations

Partially, methodological limitations refer to the fact that some papers or studies in Russian were not published as open-access and thus were not available to the international scientific community. Also, incompatible and sometimes inconsistent translations of the abstracts from Russian to English led to the mismatch between the whole content of the paper and its summary. Additionally, some authors avoided the use of the term “urban ES” or even did not accept this concept at all. The latter might be explained by the reasons provided below.

*“Theory-related reason”:* the historical background of Russian science development yielded in other terms, disciplines, and concepts of landscape functioning, anthropogenic pressure, and ecological assessment; it means that there are some well-known and well-established frameworks and approaches that can be interpreted as similar to the ES concept, but they do not use this concept directly (e.g. norms of greening in cities, landscape capacity, limits of environmental impacts, etc.). Similar to ES, the term “green infrastructure” (GI) also has its equivalents in Russian. It was introduced in the international scientific literature by Little in 1995 and since then has been broadened to a “strategically planned network of natural and semi-natural areas with other environmental features, designed and managed to deliver a wide range of ES, while also enhancing biodiversity” (EC 2024). However, in Russian scientific discourse, traditionally used terms such as “ecological framework (EF)”—*ecologicheskiy karkas (*introduced by Vladimirov 1980), “nature framework”—*prirodniy karkas* (suggested by Rejmers 1990), “nature-protected framework”—*prirodookhrannij karkas* (Tishkov 1995), “nature-ecological framework” *prirodno-ekologicheskij karkas* (see Gridnev 2011) or “ecological network” (Klimanova et al. 2021) can be found most frequently. It is important to note that the term “green infrastructure” has relatively recently appeared in Russian literature and is not mentioned in official regulatory documents where more often such terms as “the system of green spaces of the city” or “green zone of the city” or “green spaces” can be discovered. As pointed out by Klimanova et al. (2021), Klimanova and Illarionova (2020) and Illarionova (2023), opposite to the ecological network, GI includes all unbuilt UGS which differ in their functionality, connectivity, and hierarchy. The ecological framework was primarily invented as a mechanism for preservation of particular natural territories and for establishing protected areas and ecological zones. Thus, it is defined as a system of natural complexes with a high biological diversity and activity and a special regime of nature management. In contrast to the ecological network/framework, the GI concept enables economic (cost) assessment of the ES projected onto various elements of GI (from the green belt of the city to the tree and shrub group in a certain space). This provides an additional opportunity for nature protection, especially during the feasibility study of new development projects, when the benefits (estimated income) from development are proportionate to the expected losses.

*“Practice-related reason”* which refers to a weak regulatory and legal framework for ES in Russian national legislation, including economic and legal mechanisms for compensating ES (Maximova 2021). As such, the term ES is used only in a few Russian political and legal documents related to environmental issues (e.g. Law “On Conservation, Sustainable Use, and Restoration of Biological Diversity” 2016); however, they only mention the need to preserve ES and to develop a market of ES. Still, the main directions of Russian state policy in the sphere of ES at the level of political and legal documents have to be improved. Thus, all practitioners (e.g. landscape architects, land planners, nature conservation professionals, and others who work under strict regulations, even if they clearly understand the values and benefits provided by nature) do not use the ES concept in their daily practice. However, as emphasized by some Russian researchers (Klimanova et al. 2023; Konstantinova et al. 2023), the task of integrating ES into the system of spatial and strategical planning at different levels remains urgent and challenging.

Finally, *“language-related reason”* and isolation of Russia as a scientific partner by the world scientific community probably because the majority of Russian scientists still mostly publish in Russian. Primarily, it can be explained by the lack of financing and absence of such strong financial mechanisms at the Russian institutions as those that exist in the Western countries (e.g. financing of open access by libraries or grants for publishing and language proof, etc.). Moreover, since the recent geopolitical situation appeared at the beginning of 2022 and the related sanctions, international collaborations with Russian scientists have been paused for an unspecified time. Thus, many research results obtained by Russian scientists have become off-limits to most foreign researchers. We can assume should these trends continue, scientific understanding of the worldwide picture in research on urban ES and the changes in their supply–demand will significantly deteriorate.

### Directions for further research and policy implications

Research on the functioning of urban ecosystems and the goods and services they provide to the citizens (as well as the factors that impact them) can enhance our understanding of dynamics in human-nature relationships and propose new ways for urban sustainable development and resilience. Future research needs to demonstrate how the use of the ES approach in cities can contribute to the development of such urban resilience and sustainability plans and policies, governance mechanisms, and management strategies to support urban sustainable development (as proposed by Daily et al. 2009; Haase et al. 2014; TEEB DE 2016; Costanza 2020; Jiang et al. 2021) that provides benefits to both people and nature. The key areas for the future research include:

- *thematically*: ecosystem disservices (even some related studies were discovered, it is important to extend the findings on negative nature impacts on human health and well-being), supporting ES (as previously mentioned, the reviewed studies often focused on regulating or cultural ES, while supporting ES appeared to be a less studied topic that in turn could contribute to the research on urban resilience by providing further insights on maintaining their integrity, functioning, and capacity to produce other ES), urban landscape heterogeneity (since it has a range of influences on ecosystem processes, ES, and thus the sustainability of urban areas);
- *spatially*: Arctic and Northern cities as well as other Russian towns (as already noted, most studies focused on the large cities, while towns remain less studied; moreover, considering the highly urbanized character of the Russian Arctic and the North, the vulnerability of their ecosystems and their crucial role in maintaining the global environment, more research on ES in these urban areas is needed); rural–urban interactions (since ES of urban areas are often imported from rural areas, cities/towns rely on rural areas to meet their demands for food, water, wood, raw materials, recreation, etc., which are basically products of rural ES);
- *methodologically*: modelling, mapping, monitoring (e.g. creating new methods and tools for ES assessment and valuation in addition to those previously mentioned, for instance, technologies for real-time ES monitoring, models of change in ES provision based on simulation, better integration of NBS concept into UBGI and the related methods of citizen-science to support ES assessment and data collection; cross-scale and scale-sensitive tools for assessment, mapping, and modelling that connect local, regional and global scales);

The key areas/priority fields for *further implementation of ES into policy and decision-making that can be addressed through strengthening the science-policy interface*:

- better integration of ES in economic processes (monetary and non-monetary evaluations of ES; market of ES);
- deeper consideration of ES analysis and assessment in environmental and nature conservation regulations, processes, initiatives, and practices;
- identification of particularly relevant urban ES for policy-makers and society and further analysis similar to the examples provided in this review that demonstrated how urban development and planning can benefit from them;
- broader testing and validation of suggested indicators for mapping and assessment of certain ES;
- further development of options for implementing results on ES research into sectoral Russian policies, strategies, management guidelines, planning instruments, and compensation mechanisms based on the ES approach;
- training local experts (incl. public authorities, policy-makers, and other practitioners) in the use of ES-related approaches and tools that contribute to a better and shared understanding of the ES concept, its implementation into UBGI planning and management practices and a broader uptake of the concept among different scientific disciplines and beyond, also through strengthening the science-policy interface.

Currently (and as also resulted from the review), Moscow and some other large Russian cities are the leaders of innovations in the field of ES research and application in Russia. We observe a rise in the number of research and science-policy cooperations in response to the requests of the authorities on ES analysis and evaluation. This has initiated changes in the related environmental regulations and strategies, also regarding the use of ES assessment in nature protection, urban planning and development (incl. UBGI), and anthropogenic impacts on ecosystems (Minprirody 2023; Department of Nature Management and Environmental Protection of the City of Moscow 2024). Additionally, a Russian Journal Ecourbanist with more than 3,000 subscribers from science, policy, and practice in the field of urban ecology publishes popular science articles, including those on urban ES, UGS, and BGI.

## Conclusion

Using a systematic review of peer-reviewed publications, this study has identified and critically analysed the emerging trends and gaps in the existing research on urban ES in Russia. We examined how the ES concept has been adopted, modified, and operationalized in the Russian scientific discourse. We found that, on the one hand, urban ES is a very popular research field that has increasingly attracted researchers from a variety of disciplines. On the other hand, there is a certain resistance to using the ES concept (a range of the ES-related terms and approaches used in Russian scientific literature have been identified, and their general patterns have been specified). Nevertheless, these Russian scientific traditions and the school of landscape research could offer significant contributions to the research on urban ES on a broader international scale. A qualitative analysis of the most frequently cited studies revealed four key challenge areas related to urban ES research in Russia: anthropogenic pressure on soils and vegetation degradation, preservation of UGS to ensure their sufficient quantity and quality, methodological approaches to urban planning to be extended through ES assessment, comprehensive assessment of and payments for ES. In light of urban population growth and urban sprawl that are evident in many Russian cities, the issue of ensuring a healthy environment and protecting UGS from the build-up processes remains urgent. Even though Russian cities have preserved extensive green areas from the Soviet era and inherited a well-established framework for UGS development as its legacy, it is important to ensure that urban areas will not be transformed by new developments or affected by the continuing built-up processes and increasing levels of technogenic pollution. This can be achieved through the integration of the ES concept and the systems of payments for ES since UGS as UBGI elements are highly efficient suppliers of regulating and supporting ES compared to artificial or semi-natural urban spaces. To ensure the provision of ES (supply) and the amount of ES that are needed for people and the economy (demand), it is necessary to protect UGS and the whole UBGI from intense recreational use, pollution, and development/construction. Thus, it allows to support capacity of urban ecosystems to produce ES and ensure the sustainability of ES use.

## Supplementary Information

Below is the link to the electronic supplementary material.

Supplementary file1 (PDF 1797 kb)

## Data Availability

Data will be made available on request.

## Footnotes

1 www.mojgorod.ru

2 http://cyberleninka.ru.

3 https://eLibrary.ru.
