# Supplementary material for: Urban ecosystem services research in Russia: Systematic review on the state of the art
Source: Ambio. 2024 Nov 23;54(4):577–602. doi: 10.1007/s13280-024-02102-8 (PMC11871187; doi:10.1007/s13280-024-02102-8)
Supplement: Supplementary file 1 — Supplementary file1 (PDF 1797 kb) [file 13280_2024_2102_MOESM1_ESM.pdf]

Urban ecosystem services research in Russia: Systematic review on the state of the art

Supplementary material

[1,2] Diana Dushkova, [2]Anastasia Konstantinova, [2,3]Victor Matasov, [4]Dara Gaeva, [2] Elvira Dovletyarova, [2]Mina Taherkhani

[1]Department of Conservation Biology and Social-Ecological Systems and Department of Urban and Environmental Sociology, Helmholtz-Centre for Environmental Research (UFZ), Permoser str. 15, 04318, Leipzig, Germany

[2]Agrarian and Technological Institute, Peoples Friendship University of Russia (RUDN University), Miklukho-Maklaya str., 6, Moscow, Russia, 117198

[3]Faculty of Geography and Geoinformation Technology, Higher School of Economics (HSE University), 11 Pokrovsky Boulevard, Moscow, Russia, 109028

[4] Immanuel Kant Baltic Federal University, A. Nevskogo 14, Kaliningrad, Russia, 236016

Corresponding author: diana.dushkova@ufz.de

Supplementary material S1. Keywords used within searching process

Keywords	where	N of papers	The most common keywords in Russian	Russian equivalents for search
"urban ecosystem services"<br><br>*in keywords, title, and abstract*	in keywords, title, and abstract	Total – 806<br><br>In Russian - 67	**Ecosystem services:**<br><br>Экосистемные услуги (Ekosistemnye uslugi)<br><br>Урбоэкосистемные услуги (Urboekosistemnye uslugi)<br><br>Экосистемные сервисы (Ekosistemnye servisy)<br><br>Экосистемные ресурсы (Ekosistemnye resursy)<br><br>Экосистемные функции (Ekosistemnye funkcii)<br><br>**Urban:**<br><br>город(ов) / городск(ой) (gorod(ov) / gorodsk(oj))<br><br>городские территор(ии) (gorodskie territor(ii))<br><br>городская среда (gorodskaya sreda)<br><br>городской ландшафт (gorodskoj landshaft)<br><br>Урболандшафт (urbolandshaft)<br><br>Урбоэкосистема (urboekosistema)<br><br>Урбосистема (urbosistema)<br><br>урбанизированные экосистемы (urbanizirovannye ekosistemy)<br><br>урбанизированн(ые) ландшафт(ы) (urbanizirovann(ye) landshaft(y))	"Экосистемные услуги" (Ekosistemnye uslugi) AND "город*" (gorod)<br><br>"Экосистемные сервисы" (Ekosistemnye servisy) AND "город*" (gorod)<br><br>"Экосистемные функции" (Ekosistemnye funkcii) AND "город*" (gorod)<br><br>"Экологические функции" (Ekologicheskie funkcii) AND "город*" (gorod)<br><br>Урбоэкосистемные услуги (Urboekosistemnye uslugi)<br><br>"Экологические функции" (Ekologicheskie funkcii) AND "урбоэкосистем*" (urboecosystem)<br><br>"Экосистемные услуги" (Ekosistemnye uslugi) AND "урболандшафт*" (urbolandshaft)
"ecosystem services"+ "city/ies"<br><br>*in keywords, title, and abstract*	in keywords, title, and abstract	Total – 574<br><br>In Russian - 102	**Ecosystem services:**<br><br>Экосистемные возможности<br><br>Экосистемные услуги (Ekosistemnye uslugi)<br><br>Урбоэкосистемные услуги (Urboekosistemnye uslugi)<br><br>Экосистемные сервисы (Ekosistemnye servisy)<br><br>Экосистемные ресурсы (Ekosistemnye resursy)<br><br>Экосистемные возможности (Ekosistemnye vozmozhnosti)<br><br>**City/ies:**<br><br>город(ов) / городск(ой) (gorod(ov)/gorodsk(oj))<br><br>городские территор(ии) (gorodskie territor(ii))<br><br>городская среда (gorodskaya sreda)<br><br>городской ландшафт (gorodskoj landshaft)	

Keywords	where	N of papers	The most common keywords in Russian	Russian equivalents for search
			Урболандшафт (urbolandshaft)<br>Урбоэкосистема (urboekosistema)<br>Урбосистема (urbosistema)<br>урбанизированные экосистемы (urbanizirovannye ekosistemy)<br>урбанизированн(ые) ландшафт(ы) (urbanizirovann(ye) landshaft(y))	
"Ecosystem services"+ "urban" *in keywords, title, abstract, and references*	in keywords, title, abstract, and references	Total – 2941<br>In Russian - 198	**Ecosystem services:**<br>Экосистемные услуги (Ekosistemnye uslugi)<br>Урбоэкосистемные услуги (Urboekosistemnye uslugi)<br>Экосистемные сервисы (Ekosistemnye servisy)<br>Экологические функции (Ekologicheskie funkcii)<br>**Urban:**<br>город(ов) / городск(ой) (gorod(ov) / gorodsk(oj))<br>городские территор(ии) (gorodskie territor(ii))<br>городская среда (gorodskaya sreda)<br>городской ландшафт (gorodskoj landshaft)<br>Урболандшафт (urbolandshaft)<br>Урбоэкосистема (urboekosistema)<br>Урбосистема (urbosistema)<br>урбанизированные экосистемы (urbanizirovannye ekosistemy)<br>урбанизированн(ые) ландшафт(ы) (urbanizirovann(ye) landshaft(y))	

Supplementary material S2: List of Papers used for Review

1. Aleksandriiskaia, K., Klimanova, O., 2019. The assessment of recreational ecosystem services: main approach and first results, in: Biodiversity and Ecosystem Services – Management Principles in the Russia and International Processes: International Conference (Moscow, November 19–20, 2019). Moscow: Biodiversity Conservation Center Publishers, pp. 7-8.
2. Aleksandriiskaia, K., Klimanova, O., 2021. Assessing the Proposed Volume of Recreational Ecosystem Services: A Case Study of Moscow's Urban Protected Areas, in: Advanced Technologies for Sustainable Development of Urban Green Infrastructure: Proceedings of Smart and Sustainable Cities 2020. Cham: Springer, pp. 230-237. DOI 10.1007/978-3-030-75285-9_22.
3. Aleksandriiskaia, K., Klimanova, O., 2020. Environmental and recreational ecosystem services of urban protected areas: case study of Moscow in: Mirovaia ekologicheskaia povestka i Rossiia : Materialy Vserossiiskoi nauchnoi onlain-konferentsii s mezhdunarodnym uchastiem, Moskva, 16–18 noiabria 2020 goda. Moskva: Moskovskii gosudarstvennyi universitet im. M.V. Lomonosova, pp. 191-195. (in Russian)
4. Alekseeva, I., Menshikh, D., Kudryavtseva, O.V., 2016. Greening as an Element of Sustainable Urban Development: Valuation of Economic Feasibility, Policyassessment and Practical Examples. RUDN Journal of Agronomy and Animal Industries 4, 51-62. DOI 10.22363/2312-797X-2016-4-51-62.
5. Ananyeva, N.D., Ivashchenko, K.V., Sushko, S.V., 2021. Microbial indicators of urban soils and their role in the assessment of ecosystem services: a review. Eurasian Soil Science 54, 10, 1517-1531. DOI 10.31857/S0032180X21100038
6. Artamonov, G.E., Vasenev, I.I., Gutnikov, V.A., Erofeeva V.V., 2021. Environmental Assessment of Thermal Energy Facilities Impact on Ecosystem Services for the Production of Oxygen in Urban Settlements, in: Advanced Technologies for Sustainable Development of Urban Green Infrastructure : Proceedings of Smart and Sustainable Cities 2020. Cham : Springer, pp. 272-282. DOI 10.1007/978-3-030-75285-9_26.
7. Atalikhova, A.M., Pakina, A.A., 2015. integrated assessment of social environmental factors of the development of transport system of the Astana city. Ekologiia urbanizirovannykh territorii 1, 37-42. (in Russian)
8. Avershina, A.P., Karimov, E.B., Kornilov, A.G., 2020. Forests as supporting elements of the ecological framework of big cities. Geologiia, geografiia i globalnaia energeia 1(76), 113-118. (in Russian)
9. Avilova, K., 2019. The natural complex of Moscow and the activities of the Moscow city Society for Nature Defense for its protection, in: Biodiversity and Ecosystem Services – Management Principles in the Russia and International Processes: International Conference (Moscow, November 19–20, 2019). – Moscow: Biodiversity Conservation Center Publishers, pp. 8-10.
10. Avilova, K.V., Kiyatkina, N.P., 2019. Bioeconomic aspects of the city's ecosystem services on the example of the role of the nightingale (Luscinia Luscinia). Biulleten Moskovskogo obshchestva ispytatelei prirody. Otdel biologicheskii 124(4), 3-9. (in Russian)
11. Balakin, V.V., Sidorenko, V.F., Slesarev, M.Yu., Antyufeev A.V., 2019. Formation of environmental protection landscaping facilities in urban ecological systems. Vestnik MGSU 14(8), 1004-1022. DOI 10.22227/1997-0935.2019.8.1004-1022. (in Russian)
12. Balashova, E., Sharipova S., 2018. Impact of ecosystem services on a sustainable business strategy in urban conditions, in: MATEC Web of Conferences, St. Petersburg, December 20–22, 2017. St. Petersburg: EDP Sciences, 01012. DOI 10.1051/matecconf/201817001012.
13. Baranova, O.Iu., Semeniuk, O.V., 2021. Rol osobo okhraniaemykh prirodnykh territorii v podderzhanii kachestva gorodskoi sredy, in: O.P. Lavrova (eds.) Landshaftnaia arkhitektura i formirovanie komfortnoi gorodskoi sredy : Materialy KhVII regionalnoi nauchno-prakticheskoi

konferentsii, Nizhnii Novgorod, 25 marta 2021 goda. Nizhnii Novgorod: Nizhegorodskii gosudarstvennyi arkhitekturno-stroitelnyi universitet, pp. 17-24. (in Russian)

14. Baranova, O.Yu., Semenyuk, O.V., 2018. Environmental approaches and features of design for green specially protected natural urban areas. Akademicheskii vestnik UralNIIproekt RAASN 4(39), 22-27. (in Russian)
15. Bobylev, N., 2018. Gecoystem and ecosystem services - Exploring opportunitiep for inclusion in Urban underground space planning, in: ACUUS 2018 - 16th World Conference of the Associated Research Centers for the Urban Underground Space: Integrated Underground Solutions for Compact Metropolitan Cities, Conference Proceedings. Hong Kong, pp. 238-248.
16. Bobylev, S.N., Porfiriev, B.N., 2016. Sustainable development of largest cities and megalopolises: a factor of ecosystem services. Vestnik Moskovskogo universiteta. Seriia 6: Ekonomika 6, 3-21. (in Russian)
17. Bodrov, K.S., Yakovlev A.S., Semenyuk, O.V., 2016. Evaluation of ecosystem services of special protected areas and parks. Ispolzovanie i okhrana prirodnykh resursov v Rossii 2(146), 62-67. (in Russian)
18. Bodrov, K.C., Yakovlev, A.C., Semenyuk, O.V., Stoma, G.V., 2018. Methodical approaches to the economic evaluation of ecosystem services by the example of the Moscow natural park "Bitsevsky forest", in: Zdorovye pochvy - garant ustoichivogo razvitiia : Sbornik materialov nauchno-prakticheskoi konferentsii s mezhdunarodnym uchastie. Kursk: Kurskii gosudarstvennyi universitet, pp. 82-88. (in Russian)
19. Brianskaia, I.P., Vasenev, V.I., Brykova, R.A., Markelova, V. N., Ushakova, N. V., Gosse, D. D., . . . Blagodatskaya, E. V., 2020. Analysis of Volume and Properties of Imported Soils for Prediction of Carbon Stocks in Soil Constructions in the Moscow Metropolis. Eurasian Soil Science 53 (12), 1809-1817. DOI 10.1134/S1064229320120042.
20. Butserova, O.V., Golubeva, E.I., 2019. Ecological and economic assessment of the green spaces reconstruction in the territory of the National Research University of Electronic Technology (MIET) campus. Problemy regionalnoi ekologii 6, 96-103. DOI 10.24411/1728-323X-2019-18096 (in Russian)
21. Chernyshenko, O.V., Frolova V.A., Zhdanova, L.P., 2021. UN strategy and ecosystem sustainability indicators for preserving Moscow's urban biodiversity. Forestry Bulletin 25(3), 93-102. DOI 10.18698/2542-1468-2021-3-93-102 (in Russian)
22. Debelaya, I.D., Morozova, G.YU., 2020. Urban protected areas in green infrastructure of Khabarovsk city. Theoretical and Applied Ecology 3, 203-209. DOI 10.25750/1995-4301-2020-3-203-209.
23. Dovletyarova, E.A., Mosina, L.V., Stolyarova, A.G., 2012. Influence of an urban environment on pollution of soils by heavy metals depending on structure and age of wood forest stands (on an example of wood skilled giving Department of ecology Russian state agrarian university - MTAA named after K.A. Timiryazev). Vestnik Rossiiskogo universiteta druzhby narodov. Seriia: Agronomiia i zhivotnovodstvo S5, 92-98. (in Russian)
24. Dubenok, N.N., Kuzmichev, V.V., Lebedev, A.V., 2018. Growth and productivity of pine and larch stands under conditions of urban environment. Vestnik Povolzhskogo gosudarstvennogo tekhnologicheskogo universiteta. Seriia: Les. Ekologiia. Prirodopolzovanie 1(37), 54-71. DOI 10.15350/2306-2827.2018.1.54 (in Russian)
25. Dubenok, N.N., Kuzmichev, V.V., Lebedev, A.V., 2019. Ecological functions of forest stands in urbanized environment of Moscow. RUDN Journal of Agronomy and Animal Industries Vol. 14, 2, 154-161. DOI 10.22363/2312-797X-2019-14-2-154-161
26. Dubrovskaya, S.A., Ryakhov, R.V., 2020. Landscape-ecological zoning of the Orenburg city based on geomorphometric, ecological and economic features of the territory. Geoinformatika 4, 63-70. (in Russian)

27. Dubynina, S.S., Naprasnikova, E.V., 2009. Sostoianie pochv i rastitelnosti goroda Sharypovo. Geografiia i prirodnye resursy 4, 50-55. (in Russian)
28. Dushkova, D., Haase, D., Haase, A., 2016. Urban green space in transition: Historical parks and soviet heritage in Arkhangelsk, Russia. Critical Housing Analysis 3(2), 61-70. DOI 10.13060/23362839.2016.3.2.300.
29. Dushkova, D.O., Haase, D., Evseev, A.V., 2015. Ecosystem services assessment and its impact on human health - a comparative analysis of expertise and approaches in Russian and German cities. Ekologiia urbanizirovannykh territorii 4, 21-27. (In Russian)
30. Dushkova, D.O., Kirillov, S.N., 2016. Urban green infrastructure: German experience. Vestnik Volgogradskogo gosudarstvennogo universiteta. Seriia 3: Ekonomika. Ekologiia 2(35), 136-147. DOI 10.15688/jvolsu3.2016.2.14 (in Russian)
31. Dushkova, D., Ignatieva, M., Konstantinova, A., Yang F., 2021. Cultural Ecosystem Services of Urban Green Spaces. How and What People Value in Urban Nature?, in: Advanced Technologies for Sustainable Development of Urban Green Infrastructure : Proceedings of Smart and Sustainable Cities 2020. Cham : Springer, pp. 292-318. DOI 10.1007/978-3-030-75285-9_28.
32. Dvinskikh, S.A., Larchenko, O.V., 2019. Ecological characteristic of specially protected natural area of local significance "Utinoye boloto" ("Duck swamp"), Perm. Vestnik Udmurtskogo universiteta. Seriia Biologiia. Nauki o Zemle 29(1), 63-70. (in Russian)
33. Dzhus, P.O., Khanbabaeva, O.E., 2020. Gorodskoe lesopolzovanie kak osnova ustoichivogo razvitiia sredy, in: Sovremennye problemy ozeleneniia gorodskoi sredy : materialy natsionalnoi (vserossiiskoi) nauchno-prakticheskoi studencheskoi konferentsii, Novosibirsk, 23 aprelia 2020 goda. Novosibirskii gosudarstvennyi agrarnyi universitet. Novosibirsk: ITs NGAU «Zolotoi kolos», pp. 52-55. (in Russian)
34. Emuzova, L.Z., Aksorova, K.H., 2017. Ecosystem capabilities of the culture and holiday park of Nalchik "Atazhukinskaya garden", in: Ratsionalnoe prirodopolzovanie: traditsii i innovatsii : Materialy II Mezhdunarodnoi konferentsii, Moskva, 17–18 noiabria 2017 goda. Moskva: KDU, pp. 277-280. (in Russian)
35. Erokhova, V.V., Vasenev, V.I., 2018. Prospects of use of ecosystem services for estimation of scenarios of urban development. Vestnik Rossiiskogo universiteta druzhby narodov. Seriia: Agronomiia i zhivotnovodstvo 13(2), 113-120.
36. Fedorova, V.A., Safina, G.R., Alekseev, S.A., 2017. Quality of urban environment by the eyes of millenials (on the example of cities of the republic of Tatarstan). Upravlenie ustoichivym razvitiem 4(11), 75-81. (in Russian)
37. Fomenko, G.A., Fomenko, M.A., Loshadkin K.A., Mikhailova, A.V., 2019. Accounting and assessment of ecosystem services of Novokuznetsk coal-mining area (Kemerovo oblast). Izvestiia Rossiiskoi akademii nauk. Seriia geograficheskaia 3, 88-97. DOI 10.31857/S2587-55662019388-97 (in Russian)
38. Fomenko, G.A., 2020. Spatial design and ecosystem services. Problemy regionalnoi ekologii 1, 60-73. DOI 10.24411/1728-323X-2020-11060 (in Russian)
39. Frolova, V., Chernyshenko, O., Batarin, A., 2021. Use of LiDAR technology for quantification and design of park, garden and urban tree structure. IOP Conf. Series: Earth and Environmental Science 806, 012011, 1-9. doi:10.1088/1755-1315/806/1/012011
40. Galeeva, A., Mingazova, N., Gilmanshin, I., 2014. Sustainable urban development: Urban green spaces and water bodies in the city of Kazan, Russia. Mediterranean Journal of Social Sciences, 5 (24), 356-360. DOI: 10.5901/mjss.2014.v5n24p
41. Garitskaya, M.Y., Chekmareva, O.V., 2015. Environmental characteristics of various functional zones in Orenburg. Vestnik Orenburgskogo gosudarstvennogo universiteta 10(185), 371-374. (in Russian)
42. Goncharova, O.Y., Matyshak, G.V., Udovenko, M.M., Bobrik, A.A., Semenyuk, O.V., 2019. Seasonal and Annual Variations in Soil Respiration of the Artificial Landscapes (Moscow Botanical Garden),

in: Vasenev, V., Dovletyarova, E., Cheng, Z., Prokof'eva, T., Morel, J., Ananyeva, N. (eds) Urbanization: Challenge and Opportunity for Soil Functions and Ecosystem Services. SUITMA 2017. Springer Geography. Springer, Cham. https://doi.org/10.1007/978-3-319-89602-1_15

43. Ignatieva, M., Haase, D., Dushkova, D., Haase, A., 2020. Lawns in Cities: From a Globalised Urban Green Space Phenomenon to Sustainable Nature-Based Solutions. Land 9, 73. doi:10.3390/land9030073
44. Illarionova O., 2019. Air purification ecosystem services of suburban forest in the European part of Russia, in: Biodiversity and Ecosystem Services – Management Principles in the Russia and International Processes: International Conference (Moscow, November 19–20, 2019). Moscow: Biodiversity Conservation Center Publishers, pp. 22-24.
45. Illarionova, O.I., Klimanova, O.A., Kolbovsky, Yu., 2021. Regulating Ecosystem Services in Russian Cities: Can Urban Green Infrastructure Cope with Air Pollution and Heat Islands? Springer Geography, 51-64. DOI:10.1007/978-3-030-75285-9_6
46. Illarionova, O.A., Klimanova, O.A. Transformation of green infrastructure in large cities of south America. Vestnik Moskovskogo universiteta. Seriia 5: Geografiia 3, 23-29. (in Russian)
47. Illarionova, O., Klimanova, O., 2020. Green infrastructure of urban riparian zones: a literature review on assessing the state and ecosystem services in: Mirovaia ekologicheskaia povestka i Rossiia : Materialy Vserossiiskoi nauchnoi onlain-konferentsii s mezhdunarodnym uchastiem, Moskva, 16–18 noiabria 2020 goda. Moskva: Moskovskii gosudarstvennyi universitet im. M.V. Lomonosova, pp. 215-219. (in Russian)
48. Ilyichev, V.A., Kolchunov, V., Gordon, V.A., Kormina, A.A., 2021. Statistical relationships between indicators of favourable living environments in biosphere compatible cities. Vestnik MGSU 16(5), 545-556. DOI 10.22227/1997-0935.2021.5.545-556 (in Russian)
49. Insarov, G.E., Insarova, I.D., 2013. Lichens and Plants in Urban Environment. In: Malkinson, D., Czamanski, D., Benenson, I. (eds) Modeling of Land-Use and Ecological Dynamics. Cities and Nature. Springer, Berlin, Heidelberg, pp. 167-193. https://doi.org/10.1007/978-3-642-40199-2_9
50. Ivanchuk, M.S., 2009. Technique of an estimation of defense function urban plants. Vestnik Rossiiskogo universiteta druzhby narodov. Seriia: Ekologiia i bezopasnost zhiznedeiatelnosti 2, 36-40.
51. Kalmanova, V., Sukhoveeva, A., 2014. Choice and justification the system of urban environment quality indicators. Izvestiia Samarskogo nauchnogo tsentra Rossiiskoi akademii nauk 16(5-2), 878-882. (in Russian)
52. Kalmanova, V.B., 2015. Ecological-functional zoning of Birobidzhan: problems and prospects. Regionalnye problemy 18(4), 66-69. (in Russian)
53. Kartashova, N.P., Khazova, E.P., 2019. General improvement and landscaping of industrial areas on the example of NPK NIVA LLC production centre, Krasnodar. Lesotekhnicheskii zhurnal 9, 3(35), 45-55. DOI 10.34220/issn.2222-7962/2019.3/5 (in Russian)
54. Kataeva, Y.V., Lapin, A.V., 2014. Developing of methodical approach to urban space integrated estimation. Vestnik Permskogo universiteta. Seriia: Ekonomika 2(21), 31-39. (in Russian)
55. Kazarian, R.A., 2021. The development of modern cities from the perspective of an ecological approach. Ekonomika stroitelstva i prirodopolzovaniia 1(78), 28-34. DOI 10.37279/2519-4453-2021-1-28-34 (in Russian)
56. Khanbabaeva, O.E., Dzhus, P.O., 2020. Prospects for Greening Urban Areas, in: E.I. Antonova (eds.) Fundamentalnye i prikladnye issledovaniia po prioritetnym napravleniiam bioekologii i biotekhnologii : Sbornik materialov III Vserossiiskoi nauchno-prakticheskoi konferentsii, Ulianovsk, 20 maia 2020 goda. Ulianovsk: Obshchestvo s ogranichennoi otvetstvennostiu «Izdatelskii dom «Sreda», pp. 86-90. DOI 10.31483/r-75367 (in Russian)
57. Khnykina, A.S., 2018. Drevesnye rasteniia v sostave gorodskikh nasazhdenii: osobennosti sostoianiia i effektivnost v okazanii ekosistemnykh uslug, in: XX Vserossiiskaia studencheskaia nauchno-prakticheskaia konferentsiia Nizhnevartovskogo gosudarstvennogo universiteta :

sbornik statei, Nizhnevartovsk, 03–04 aprelia 2018 goda / Otvetstvennyi redaktor A.V. Korichko. Nizhnevartovsk: Nizhnevartovskii gosudarstvennyi universitet, pp. 61-64. (in Russian)
58. Khromova, T.M., Knyazev, S.D., Emelyanova, O.Yu., Zolotareva, E.V., 2019. Eco-floristic monitoring in the urbanized territories on the example of the Orel region' cities. Biulleten Gosudarstvennogo Nikitskogo botanicheskogo sada 133, 17-23. DOI 10.36305/0513-1634-2019-133-17-23 (in Russian)
59. Kirpichev, I.A., Savvateeva, O.A., Dzhamalov, R.G., Starostin, E.A., 2020. Ecological state of surface water in dubna city as one of the factors of impact on environment and population health. Uspekhi sovremennogo estestvoznaniia 12, 85-91. DOI 10.17513/use.37542 (in Russian)
60. Kisova, S.V., Tatarnikova, V.YU., Kazakov, M.V., Pashina, M.N., 2021. Landscaping as a factor of formation pleasant human environment on example of public use objects in Oktyabrskiy district of Ulan-Ude. Vestnik Buriatskoi gosudarstvennoi selskokhoziaistvennoi akademii im. V.R. Filippova 2(63), 92-99. DOI 10.34655/bgsha.2021.63.2.013 (in Russian)
61. Kleimenov, T.V., 2008. Ekologicheskie informatsionnye sistemy v upravlenii monitoringom zelenoi zony goroda. Uspekhi sovremennogo estestvoznaniia 2, 124-125. (in Russian)
62. Klimanova, O.A., Illarionova, O.I., 2020. Green infrastructure indicators for urban planning: applying the integrated approach for Russian largest cities. Geography, Environment, Sustainability 13(1), 251-259. DOI: 10.24057/2071-9388-2019-123
63. Klimanova, O.A., Kolbovsky, E.Yu., Illarionova, O.A., 2018. The ecological framework of Russian major cities: Spatial structure, territorial planning and main problems of development. Vestnik of Saint Petersburg University. Earth Sciences 63(2), 127-146. DOI 10.21638/11701/spbu07.2018.201.
64. Klimanova, O.A., Kolbovskii, E.Y., Kurbakovskaya, A.V., 2016. Assessing the geoecological functions of the green infrastructure in cities of Canada. Geography and Natural Resources 37(2), 165-173.
65. Klimanova, O.A., Titova, L.A., 2020. Green infrastructure transformation in the post-soviet capitals of Central Asia, in: Mirovaia ekologicheskaia povestka i Rossiia : Materialy Vserossiiskoi nauchnoi onlain-konferentsii s mezhdunarodnym uchastiem, Moskva, 16–18 noiabria 2020 goda. Moskva: Moskovskii gosudarstvennyi universitet im. M.V. Lomonosova, pp. 219-223. (in Russian)
66. Klimanova, O., Illarionova, O., 2019. Mapping and assessment of ecosystem services in Russia's largest cities: the first results, in: Biodiversity and Ecosystem Services – Management Principles in the Russia and International Processes: International Conference (Moscow, November 19–20, 2019). Moscow: Biodiversity Conservation Center Publishers, pp. 28-30.
67. Klimanova, O., Illarionova, O., Grunewald, K., Bukvareva, E., 2021. Green Infrastructure, Urbanization, and Ecosystem Services: The Main Challenges for Russia's Largest Cities. Land 10, 1292. doi:10.3390/land10121292
68. Klimanova, O., Kolbovsky, E., Illarionova, O., 2018. Impacts of urbanization on green infrastructure ecosystem services: The case study of post-soviet Moscow. BELGEO 4, 1-15. DOI 10.4000/BELGEO.30889.
69. Kochurov, B.I., Ivashkina, I.V., 2015. The role of open spaces in maintaining the ecological balance in the large city. Ekologiia urbanizirovannykh territorii 1, 6-13. (in Russian)
70. Kolbowsky, E.Yu., Petrov, L.A., 2020. Assessment of park aquatic complexes in the context of GIS modelling of the visual aesthetic properties of the territory of Moscow, in: Mirovaia ekologicheskaia povestka i Rossiia : Materialy Vserossiiskoi nauchnoi onlain-konferentsii s mezhdunarodnym uchastiem, Moskva, 16–18 noiabria 2020 goda. Moskva: Moskovskii gosudarstvennyi universitet im. M.V. Lomonosova, pp. 276-281. (in Russian)
71. Komarchev, D.A., Krasovskaya, T.M., 2020. Comparative characteristic of urban green infrastructure ecosystem services in temperate and subarctic zones, in: A. Fedorova (eds.) Ekologicheskie problemy. Vzgliad v budushchee : sbornik trudov IX Mezhdunarodnoi nauchno-

praktícheskoi konferentsii, Rostov-na-Donu, 22–23 oktiabria 2020 goda. Iuzhnyi federalnyi universitet. Rostov-na-Donu - Taganrog: Iuzhnyi federalnyi universitet, pp. 351-355. (in Russian)

72. Komissarov, V.N., Sibagatova, E.R., Yavorsky, A.S., 2020. Green plantations as ecological framework the urbanized territories, in: Studencheskaia nauka - agrarnomu proizvodstvu : Materialy 78-oi studencheskoi (regionalnoi) nauchnoi konferentsii, Kazan, 27 fevralia 2020 goda. Tom 3. – Kazan: Kazanskii gosudarstvennyi agrarnyi universitet, pp. 61-63. (in Russian)
73. Koposova, N.N., Shaybekova, M.R., 2017. Design park "Mesherskoe lake" as structural part of ecological frame of Nizhny Novgorod city. Uspekhi sovremennogo estestvoznaniia 7, 81-85. (in Russian)
74. Korendiaseva, E.V., 2015. Ekologicheskoe blagoustroistvo goroda kak osnova komfortnogo prozhivaniia moskvichei. Vestnik MGUU 1, 48-51. (in Russian)
75. Korlyakov, I., Kasimov, N., Kosheleva, N., 2019. Heavy metals and metalloids in the soil cover of the city of Ulan-Ude. Vestnik Permskogo natsionalnogo issledovatelskogo politekhnicheskogo universiteta. Prikladnaia ekologiia. Urbanistika 3(35), 120-137. DOI 10.15593/2409-5125/2019.03.09 (in Russian)
76. Kozlova, A.A., Makarova, A.P., Ivanuta, L.A., Vashkevich, N.V., 2006. ecological function of Irkutsk urban soils. Biulleten Vostochno-Sibirskogo nauchnogo tsentra Sibirskogo otdeleniia Rossiiskoi akademii meditsinskikh nauk 2(48), 50-56. (in Russian)
77. Kozlova, D., 2020. Sokolniki park: historical value and ecosystem functions. StudArctic Forum 1(17), 11-16. – DOI 10.15393/j102.art.2020.4601 (in Russian)
78. Krupina, N.N., 2017. “Live” fixed assets (perennial plantations) as an object of economic analysis. Ekonomicheskii analiz: teoriia i praktika 16, 11(470), 2043-2063. DOI 10.24891/ea.16.11.2043. (In Russian)
79. Kusova, N.Kh., 2016. Zelenye nasazhdeniia - osnovnoi komponent ekologicheskoi sistemy Vladikavkaza. Izvestiia Chechenskogo gosudarstvennogo pedagogicheskogo universiteta Seriia 2. Estestvennye i tekhnicheskie nauki 10, 2(14), 22-27. (in Russian)
80. Kuzmichev, V., Lebedev, A., Dubenok, N., 2018. Analysis of ecological functions of birch and oak stands in conditions of urbanized environment on materials of long-term observations. Rossiiskaia selskokhoziaistvennaia nauka 5, 29-31. DOI 10.31857/S250026270000632-0 (in Russian)
81. Li, M.Y., 2020. Assessment of urban ecosystem services, in: Mirovaia ekologicheskaia povestka i Rossiia : Materialy Vserossiiskoi nauchnoi onlain-konferentsii s mezhdunarodnym uchastiem, Moskva, 16–18 noiabria 2020 goda. Moskva: Moskovskii gosudarstvennyi universitet im. M.V. Lomonosova, pp. 230-234. (in Russian)
82. Lisina, N.L., 2015. Current status and problems of the urban environment. Vestnik Kemerovskogo gosudarstvennogo universiteta 4-2(64), 242-246. (in Russian)
83. Loshakov, A., 2017. Features of composition and structure of soil microbial community on the example of the recreational zone of megapolis. Aktualnye nauchnye issledovaniia v sovremennom mire 4-7(24), 63-67. (in Russian)
84. Lykov, I.N., Loboda, N.B., 2019. Ecological and medical-biological significance of landscape and relief. Problemy regionalnoi ekologii 2, 12-16. DOI 10.24411/1728-323X-2019-12012. (in Russian)
85. Maltseva, I.N., Kaganovich, N.N., Mindiyrova, T.N., 2017. Agricultural Urbanism in the Context of Landscape Ecological Architecture. IOP Conference Series: Materials Science and Engineering, Chelyabinsk, Vol. 262. Chelyabinsk: Institute of Physics Publishing, 012151. DOI 10.1088/1757-899X/262/1/012151.
86. Markelova, S.A., Tikhonova, I.O., 2017. The importance of ecosystem services in the sustainable development of megacities on the example of water, in: Ekologicheskaia, promyshlennaia i energeticheskaia bezopasnost - 2017 : Sbornik statei po materialam nauchno-prakticheskoi konferentsii s mezhdunarodnym uchastiem, Sevastopol, 11–15 sentiabria 2017 goda / Pod redaktsiei Iu.A. Omelchuk, N.V. Liaminoi, G.V. Kucherik. Sevastopol: Federalnoe gosudarstvennoe

avtonomnoe obrazovatelnoe uchrezhdenie vysshego obrazovaniia "Sevastopolskii gosudarstvennyi universitet", pp. 846-849. (in Russian)
87. Markelova, S.A., Tikhonova, I.O., 2017. Applicability of the concept of "ecosystem services" for water objects in the city. Uspekhi v khimii i khimicheskoi tekhnologii 31, 9(190), 68-70. (in Russian)
88. Martynova, N.A., Pushkareva, V.S., 2019. Soil resource and ecological potential of forest landscapes of Irkutsk-city and its surroundings. Pochvy i okruzhaiushchaia sreda 2(1), 1-14. DOI 10.31251/pos.v2i1.48 (in Russian)
89. Matasov, V., Belelli Marchesini, L., Yaroslavtsev, A., Sala, G., Fareeva, O., Seregin, I., Castaldi, S., Vasenev, V., Valentini, R., 2020. IoT Monitoring of Urban Tree Ecosystem Services: Possibilities and Challenges. Forests 11, 775. doi:10.3390/f11070775
90. Matasov, V., Yaroslavtsev, A., Bukin, S., Konstantinov, P., Vasenev, V., Grigoreva, V., Maximova, O., 2021. Ecosystem Services Approach for Landscaping Project: The Case of Metropolia Residential Complex, in: Advanced Technologies for Sustainable Development of Urban Green Infrastructure : Proceedings of Smart and Sustainable Cities 2020. Cham : Springer, pp. 319-330. DOI 10.1007/978-3-030-75285-9_29
91. Matova, N.I.,2018. Formy obshchestvennogo uchastiia v formirovanii OOPT mestnogo znacheniia kak elementa "zelenoi" infrastruktury ustoichivogo goroda, in: Ustoichivoe razvitie osobo okhraniaemykh prirodnykh territorii : Sbornik statei V Vserossiiskoi nauchno-prakticheskoi konferentsii, Sochi, 10–12 oktiabria 2018 goda. Sochi: Gosudarstvennoe kazennoe uchrezhdenie Krasnodarskogo kraia "Prirodnyi ornitologicheskii park v Imeretinskoi nizmennosti", pp. 204-210. (in Russian)
92. Matyushkina, L.A., Kalmanova, V.B., 2016. Map support for environmental planning of a medium-size industrial city. INTERCATRO. INTERGIS 22(2), 286-300. (in Russian)
93. Medvedeva, O.E., Soloveva, S. V., Medvedev, P. V., 2016. Metodika stoimostnoi otsenki ushcherba, prichiniaemogo prirodnym kompleksam gorodov (parkov) na osnove otsenki vypolniaemykh imi ekosistemnykh uslug na primere Moskvy. Voprosy otsenki 3(85), 12-22. (In Russian)
94. Medvedeva, O.E., 2020. Cost evaluation of ecosystem services of city parks, in: Natsionalnye prioritety i bezopasnost : Sbornik nauchnykh trudov po materialam mezhdunarodnoi nauchno-prakticheskoi konferentsii. Nalchik: Federalnoe gosudarstvennoe biudzhetnoe obrazovatelnoe uchrezhdenie vysshego obrazovaniia "Kabardino-Balkarskii gosudarstvennyi agrarnyi universitet imeni V.M. Kokova", pp. 441-443. (in Russian)
95. Mezenina, O.B., Atkina, L.I., Zhukova, M.V., Lukin D.A., 2019. Assessment of environmental-economic damage as a result of anthropogenic transformation of parkland in Yekaterinburg. Moskovskii ekonomicheskii zhurnal 3, 72-83. DOI 10.24411/2413-046X-2019-13024 (in Russian)
96. Mikhailova, T.A., Shergina, O.V., 2015. Ecological criteria for calculation green plantations area within industrial cities. Uspekhi sovremennogo estestvoznaniia 6, 123-128. (in Russian)
97. Mikhaylova, I., Slukovskaya, M., Mosendz, I., Kremenetskaya, I., Karavayeva, E., Drogobuzhskaya, S., 2019. Application of Silicon-Contained Mining Wastes in Urban Greening, in: Vasenev, V., Dovletyarova, E., Cheng, Z., Prokof’eva, T., Morel, J., Ananyeva, N. (eds) Urbanization: Challenge and Opportunity for Soil Functions and Ecosystem Services. SUITMA 2017. Springer Geography. Springer, Cham. https://doi.org/10.1007/978-3-319-89602-1_18
98. Miksyuk, A.A., Ishkhanova, K., 2014. Moscow parks: between nature and entertainment, in: International Multidisciplinary Scientific Conferences On Social Sciences And Arts SGEM 2014 : Conference Proceedings, Albena, Bulgaria, Vol. II. Book 1. Albena, Bulgaria: STEF92 Technology, pp. 535-542. DOI 10.5593/sgemsocial2014/B12/S2.068
99. Mingaleva, T.A., Goryachev, A.A., Mingaleva, E.I., Mingalev, A.I., 2020. Social and environmental functions of public parks in a small northern town (on the example of the Apatity Town in the Murmansk Region). Transaction Kola Science Centre. 2. 179-195. 10.37614/2307-5252.2020.2.8.019. (in Russian)

100. Minigazimov, N.S., Khydarshina, E.T., Kuantaeva, A.A. Evaluation of pollution level of soils of the city of Ufa. Rossiiskii elektronnyi nauchnyi zhurnal 1(31), 56-72. DOI 10.31563/2308-9644-2019-31-1-56-72 (in Russian)
101. Mironova, E.E., 2020. GIS modeling of green infrastructure of Mediterranean cities for management of urbanized ecosystems. Ecosystems: Ecology and Dynamics 4(4), 37-56.
102. Mironova, E.E., 2020. GIS modeling of green infrastructure of Mediterranean cities for management of urbanized ecosystems. Ekosistemy: ekologiia i dinamika 4(4), 14-36. (in Russian)
103. Mitrakova, N.V., Belomorskaya, Y.W., Agafonova, E.V., 2016. Evaluation of soil residential and industrial zones Perm by fitotestirovaniya. Antropogennaia transformatsiia prirodnoi sredy 2, 217-222. (in Russian)
104. Mishina, A.S., Grin, I.Y., 2011. Formation of green frame of Khabarovsk historical center. Novye idei novogo veka: materialy mezhdunarodnoi nauchnoi konferentsii FAD TOGU 1, 226-230. (in Russian)
105. Mochalova, L.A., Iurak, V.V., Grinenko, D.A., 2017. Uchet ekosistemnykh uslug pri priniatii upravlencheskikh reshenii na urovne goroda, in: Aktualnye problemy ekonomiki i upravleniia : sbornik statei Piatoi zaochnoi vserossiiskoi nauchno-prakticheskoi konferentsii, Ekaterinburg, 11–12 sentiabria 2017 goda. Ekaterinburg: Uralskii gosudarstvennyi gornyi universitet, pp. 180-186. (in Russian)
106. Mokretsov, YU.V., Avdeev, YU.M., Tesalovsky, A.A., Lukashevich, V.M., 2020. Improving the legal regime and methods of compensating the harm to the environment of Russia. Pravo i gosudarstvo: teoriia i praktika 3(183), 197-199. (in Russian)
107. Mokshina, D.D., 2013. Improve sustainable green space for improvement of environmental safety. Antropogennaia transformatsiia prirodnoi sredy 1, 152-154. (in Russian)
108. Morozova, G.Yu., Debelaya, I.D., 2018. Green Infrastructure as a Factor for Sustainable Development of Khabarovsk. Economy of Region 14(2), 562-574. DOI 10.17059/2018-2-18.
109. Morozova G.Yu., Debelaia I.D., 2020. Thematic structure of the GIS database Khabarovsk city protected areas. Geodesy and cartography 81(7), 56-64. DOI: 10.22389/0016-7126-2020-961-7-56-64 (In Russian)
110. Mozolevskaia, E.G., Bednova, O.V., Rysin, S. L., 2009. "GOROD. LES. OTDYKh" Nauchnaia konferentsiia "Rekreatsionnoe ispolzovanie lesov na urbanizirovannykh territoriiakh". Vestnik Moskovskogo gosudarstvennogo universiteta lesa - Lesnoi vestnik 5, 212-216. (in Russian)
111. Nahapetyan, A.A., Erokhova, V.V., 2018. Prospects for the Introduction of Evaluation Methods in the Process of Urbanization at Different Stages. Overview. Contemporary Problems of Social Work Vol. 4, 1(13), 99-107. DOI 10.17922/2412-5466-2018-4-1-99-107.
112. Nakhapetyan, A.A., Erokhova, V.V., 2018. Analysis of the Dynamics of Changes in the Elemental Composition of Ecosystems Under the Influence of Anthropogenic Activities. Contemporary Problems of Social Work Vol. 4, 4(16), 53-58.DOI 10.17922/2412-5466-2018-4-4-53-58
113. Nastinova, G.E., Amikova, E.A., 2021. Ecological landscape approach to greening of the urban environment in arid conditions (on the example of Elista). Estestvennye i tekhnicheskie nauki 2(153), 41-48. (in Russian)
114. Nevedrov, N.P., 2018. Classification of soil damages of Kursk city ecosystems. Astrakhanskii vestnik ekologicheskogo obrazovaniia 2(44), 111-118. (in Russian)
115. Nevedrov, N., Protsenko E., 2018. Technologies for optimization of ecosystem services and functions of soils under anthropogenic impact in urban areas, in: IOP Conference Series: Earth and Environmental Science : 3. Moscow, pp. 012009. DOI 10.1088/1755-1315/177/1/012009
116. Nikitina, O.A., 2006. To the question of steady ecological-economic of an urban recreation. Uspekhi sovremennogo estestvoznaniia 4, 60. (in Russian)
117. Odnoralov, G.A., Tikhonova, E.N., Golyadkina, I.V., Malinina, T.A., 2020. Assessment of urban forest biological productivity (case study of the Voronezh upland oak forest). Izvestiia

vysshikh uchebnykh zavedenii. Lesnoi zhurnal 2(374), 60-72. DOI 10.37482/0536-1036-2020-2-60-72 (in Russian)

118. Omarova, M.N., Orakbay, L.Z., Cherepanova, L.Y., Glubokovskich, L.K., 2015. Modern aspects of comprehensive assessment of health ecological situation in the metropolitan area (analytical review). Mezhdunarodnyi zhurnal prikladnykh i fundamentalnykh issledovanii 12-5, 830-837. (in Russian)

119. Parfenyuk, T.A., Strakhova, K.A., Martsinevskaya, L.V., 2021. The role of green spaces in improving the environmental conditions of cities and towns. International Journal of Humanities and Natural Sciences 5-2(56), 36-38. DOI 10.24412/2500-1000-2021-5-2-36-38

120. Pashkevich, M.A., Bech, Ja., Matveeva, V.A., Alekseenko A.V., 2020. Biogeochemical assessment of soils and plants in industrial, residential and recreational areas of Saint Petersburg. Journal of Mining Institute 241, 1, 125-130. DOI 10.31897/PMI.2020.1.125

121. Petrov, L.A., Kolbovsky, E.YU., 2020. Modern methods of spatial analysis green infrastructure of urbanized territories (on the example of the town of Grozny). Groznenskii estestvennonauchnyi biulleten V.5, 3(21), 39-51. DOI 10.25744/genb.2020.20.3.004 (in Russian)

122. Potapova, Ye., 2016. Ecosystem services planting area settlements. Biulleten nauki i praktiki 9(10), 36-41. DOI 10.5281/zenodo.154210. (in Russian)

123. Potapova, E., 2015. Environmental risks and factors for green areas. Bezopasnost v tekhnosfere 4(6), 17-22. DOI 10.12737/17549 (in Russian)

124. Potapova, E.V., Zelinskaya, E.V., 2015. Environmental risk assessment for the urban green areas. Vestnik Severnogo (Arkticheskogo) federalnogo universiteta. Seriia: Estestvennye nauki 4, 25-34. DOI 10.17238/issn2227-6572.2015.4.25 (in Russian)

125. Potapova, E.V., 2015. Identification of environmental risks for green areas in cities. Izvestiia Irkutskogo gosudarstvennogo universiteta.Seriia: Nauki o Zemle 11, 83-94. (in Russian)

126. Potapova, E.V., 2016. The study of conditions of greenspaces in Irkutsk-2. Vestnik Udmurtskogo universiteta. Seriia Biologiia. Nauki o Zemle 26(1), 29-36. (in Russian)

127. Potapova, E.V., 2016. Significance of environmental risks of green areas of the cities. Vestnik Kurskoi gosudarstvennoi selskokhoziaistvennoi akademii 5, 76-80. (n Russian)

128. Potapova, E.V., Zelinskaya, E.V., The analysis of environmental risks for urban plot of land. Vestnik Rossiiskogo universiteta druzhby narodov. Seriia: Ekologiia i bezopasnost zhiznedeiatelnosti 4, 70-81. (in Russian)

129. Potapova, E., Kruchina, E., Barkhatova, O., 2021. Green Areas as a Framework for Ensuring the Resilient of Cities. 2021 IOP Conf. Ser.: Earth Environ. Sci. 666, 4, 042028. DOI: 10.1088/1755-1315/666/4/042028

130. Preshkin, G.A., Bezrukova, T.L., 2017. Approach to measuring the value of landscaped areas on the lands of settlements. Lesotekhnicheskii zhurnal 7, 1(25), 233-240.

131. Prokofev, I.L., Shevko, O.A., Seredina, A.P., 2013. Role of earthworms in ensuring the ecological functions of soil in the city of Bryansk. Ezhegodnik NII fundamentalnykh i prikladnykh issledovanii 1(4), 34-40. (in Russian)

132. Razinkova, A.K., Perelygina, E.N., 2016. Evaluation of green trees children's park "Eaglet" in Voronezh. Lesotekhnicheskii zhurnal 6, 1(21), 104-115. DOI 10.12737/18732. (in Russian)

133. Romanova, E.P., Arshinova, M.A., 2018. Food supplying ecosystem services in European cities. Geografiia i ekologiia v shkole XXI veka 6, 32-35.

134. Romzaykina, O. N., 2017. Ecosystem services of urban soils in functional zones of Moscow (analysis the pollution consequences), in: Modern Paradigm of the Scientific Knowledge: Actuality and Prospects. Moskva: Obshchestvo s ogranichennoi otvetstvennostiu "Iazyki Narodov Mira", pp. 24-25.

135. Romzaykina, O.N., Vasenev, V.I., Paltseva, A., Kuzyakov, Y.V., Neaman, A., Dovletyarova, E.A., 2021. Assessing and mapping urban soils as geochemical barriers for contamination by heavy

metal(loid)s in Moscow megapolis. J. Environ. Qual. 50, 22– 37. https://doi.org/10.1002/jeq2.20142
136. Rozlomiy, N.G., Gukov, G.V., 2020. Aesthetic assessment of the ecological potential of the urbanized area (on the example of forests in the green zone of the cities of the South). Uspekhi sovremennogo estestvoznaniia 8, 26-31. DOI 10.17513/use.37453 (in Russian)
137. Rumyantsev, D.E., Frolova, V.A., 2019. Methodological approaches to studying diversity of ecosystem services of greeneries in metropolitan city. Mezhdunarodnyi nauchno-issledovatelskii zhurnal 10-2(88), 28-34. DOI 10.23670/IRJ.2019.88.10.028 (in Russian)
138. Runova, E.M., Gnatkovich, P.S., 2015. Recreational Use Prospects of Urban Forests on Residential Area in Bratsk. Izvestiia vysshikh uchebnykh zavedenii. Lesnoi zhurnal 3(345), 43-52. (in Russian)
139. Russo, A., Cirella G.T., 2018. Modern compact cities: How much greenery do we need? International Journal of Environmental Research and Public Health 15, 10, 2180. DOI 10.3390/ijerph15102180
140. Russo, A., Escobedo, F.J., Cirella, G.T., Zerbe S., 2017. Edible green infrastructure: An approach and review of provisioning ecosystem services and disservices in urban environments. Agriculture, Ecosystems & Environment 242, 53-66. DOI 10.1016/j.agee.2017.03.026
141. Sarzhanov, D.A., Vasenev, I.I., Valentini R., 2015. Analiz prostranstvennogo raznoobraziia i vremennoi dinamiki pochvennykh potokov parnikovykh gazov (CO2, SH4, N2O) v usloviiakh predstavitelnykh urboekosistem g. Kurska. AgroEkoInfo 6(22). (in Russian)
142. Savetkanov, D.E., Klimanova, O.A., 2018. Metody otsenki izmenenii kachestva zelenoi infrastruktury goroda Astany s 2000 po 2017 gody, in: Indikatsiia sostoianiia okruzhaiushchei sredy: teoriia, praktika, obrazovanie : Trudy shestoi mezhdunarodnoi nauchno-prakticheskoi konferentsii: sbornik statei. Moskva: Buki-Vedi, pp. 103-108. (in Russian)
143. Savetkanov, D.E., Klimanova, O.A., 2018. Ecosystem services of elements of the green infrastructure of Astana and their geo-information assessment. Priroda i obshchestvo: v poiskakh garmonii 4, 98-109. (in Russian)
144. Semenyuk, O.V., Baranova, O.Y., Bodrov, K.S., Stoma, G.V., 2020. The use of ecosystem services and the regulatory and methodological framework in calculating the valuation of urban land, in: Otrazhenie bio-, geo-, antroposfernykh vzaimodeistvii v pochvakh i pochvennom pokrove : Sbornik materialov VII Mezhdunarodnoi nauchnoi konferentsii, posviashchennoi 90-letiiu kafedry pochvovedeniia i ekologii pochv TGU, Tomsk, 14–19 sentiabria 2020 goda. Tomsk: Izdatelskii Dom Tomskogo gosudarstvennogo universiteta, pp. 300-303. (in Russian)
145. Semenyuk, O.V., Bodrov, K.S, Stoma, G.V., Yakovlev, A.S., 2019. Estimation of the value of ecosystem services of the natural park “Bitzevsky forest”. Vestnik Moskovskogo universiteta. Seriia 17: Pochvovedenie 3, 23-30. (in Russian)
146. Semenyuk, O.V., Stoma, G.V., Bodrov, K.S., 2021. Cost evaluation of ecosystem services of urban landscapes, in: Pochva kak sviazuiushchee zveno funktsionirovaniia prirodnykh i antropogenno-preobrazovannykh ekosistem : materialy V Mezhdunarodnoi nauchno-prakticheskoi konferentsii, posviashchennoi 90-letiiu kafedry pochvovedeniia i otsenki zemelnykh resursov IGU i Dniu Baikala, Irkutsk, 23–29 avgusta 2021 goda. Irkutsk: Irkutskii gosudarstvennyi universitet, pp. 514-518. (in Russian)
147. Sergienko, L.I., Brysgalina, E.S., 2012. Town soils in conditions of technogenez on example of town Volzhskiy. Agrarnaia nauka 6, 5-7. (in Russian)
148. Shabanova, A.V., 2014. Development of a system of indicators of recreational load on urban protected areas on the example of the limnological complexes in the city of Samara. Sotsiologiia goroda 3, 17-30. (in Russian)
149. Shabanova, A.V., 2015. From agriculture to recreation: socially important functions of urban reservoirs on example of Samara, XIX-XXI centuries. Regionalnoe razvitie: elektronnyi nauchno-prakticheskii zhurnal 7(11). (in Russian)

150. Shukurov, I.S., Le, M.T., Shukurova, L.IL., Dmitrieva, A.D., 2020. Influence of the effect of the urban heat island on the cities sustainable development. Gradostroitelstvo i arkhitektura 10, 2(39), 62-70. DOI 10.17673/Vestnik.2020.02.9 (in Russian)
151. Slepnev, M.A., Zolotaykina, I.A., 2021. Sustainable development of a protected natural area on the example of the Birulevsky dendrarium in the Moscow city. Ekologiia urbanizirovannykh territorii 1, 60-67. DOI: 10.24412/1816-1863-2021-1-60-67 (in Russian)
152. Slobodchikova, N.A., Ivanilova, E,A., 2018. The question of the need for green plantations on the city streets in the Russian Federation. Molodezhnyi vestnik IrGTU 8(4), 23-26. (in Russian)
153. Sobolev, N.A., Volkova, L.B., 2019. Place of forests in the natural frame of urbanized territories, in: Aktualnye problemy ekologii i prirodopolzovaniia : sbornik nauchnykh trudov XX Mezhdunarodnoi nauchno-prakticheskoi konferentsii: v 2 t., Moskva, 25–27 aprelia 2019 goda / Rossiiskii universitet druzhby narodov. Tom 2. Moskva: Rossiiskii universitet druzhby narodov (RUDN), pp. 102-106. (in Russian)
154. Sokolova, O.E., Potapova, E.V., Barkhatova, O.A., Functions and ecosystem services of the urban green spaces, in: Geograficheskie issledovaniia Aziatskoi Rossii i sopredelnykh territorii: novye metody i podkhody : Materialy Mezhdunarodnoi konferentsii, posviashchennoi 70-letiiu geograficheskogo fakulteta IGU, Irkutsk, 01–03 oktiabria 2019 goda. Irkutsk: Irkutskii gosudarstvennyi universitet, pp. 211-215. (in Russian)
155. Sokolova, O.E., 2020. The role of ecosystem services and their functions in sustainable urban development, in: Tekhnologii i oborudovanie sadovo-parkovogo i landshaftnogo stroitelstva : Sbornik statei Mezhdunarodnoi nauchno-prakticheskoi konferentsii, Krasnoiarsk, 18 dekabria 2019 goda. – Krasnoiarsk: Federalnoe gosudarstvennoe biudzhetnoe obrazovatelnoe uchrezhdenie vysshego obrazovaniia "Sibirskii gosudarstvennyi universitet nauki i tekhnologii imeni akademika M.F. Reshetneva", pp. 298-301. (in Russian)
156. Solodov, S.V., Lugovskoy, A.M., 2019. Implementation of ecological education activities in city's parks and specially protected natural areas. Uspekhi sovremennogo estestvoznaniia 3, 98-103. (in Russian)
157. Stoma, G.V., Semenyuk, O.V., Bodrov, K.A., 2021. Ecosystem services of park landscapes and calculation their valuation, in: Tekhnologii i oborudovanie sadovo-parkovogo i landshaftnogo stroitelstva : Sbornik statei vserossiiskoi nauchno-prakticheskoi konferentsii, Krasnoiarsk, 23 dekabria 2020 goda. Krasnoiarsk: Federalnoe gosudarstvennoe biudzhetnoe obrazovatelnoe uchrezhdenie vysshego obrazovaniia "Sibirskii gosudarstvennyi universitet nauki i tekhnologii imeni akademika M.F. Reshetneva", pp. 243-245. (in Russian)
158. Stupakova, O.M., Aksianova, T.Iu., Gaponova G.A., 2011. Obosnovannost neobkhodimosti primeneniia khvoinykh porod dlia ozeleneniia territorii shkol G.Krasnoiarska. Khvoinye borealnoi zony 29, 3-4, 278-280. (in Russian)
159. Sulkarnaeva, L.D., 2018. Adaptation of ecosystem services concept for Russian cities. Uspekhi sovremennogo estestvoznaniia 12, 207-210. (in Russian)
160. Sulkarnaeva, L. D., 2016. Otsenka urboekosistemnykh uslug dlia ustoichivogo gorodskogo razvitiia (na primere goroda Tiumen). Uspekhi sovremennoi nauki 10 (11), 72-78. (In Russian)
161. Sulkarnaeva L.D., 2017. Determination of approaches to evaluating urban and ecological system services in Russian cities. Mezhdunarodnyi nauchno-issledovatelskii zhurnal 9-2(63), 80-84. DOI 10.23670/IRJ.2017.63.068. (in Russian)
162. Sulkarnaeva, L.D., 2018. Using of urban ecosystem services assessment for sustainable urban development, in: Prostranstvennaia organizatsiia obshchestva: teoriia, metodologiia, praktika : Sbornik materialov Mezhdunarodnoi nauchno-prakticheskoi konferentsii , Perm, 07–11 noiabria 2018 goda / Pod redaktsiei T.V. Subbotinoi, L.B. Chupinoi. Perm: Permskii gosudarstvennyi natsionalnyi issledovatelskii universitet, pp. 91-95. (in Russian)
163. Sulkarnaeva L.D., 2017. Algorithm for urban ecosystem services assessment for sustainable urban development, in: Landshaftovedenie: teoriia, metody, landshaftno-

ekologicheskoe obespechenie prirodopolzovaniia i ustoichivogo razvitiia : materialy XII Mezhdunarodnoi landshaftnoi konferentsii, Tiumen-Tobolsk, 22–25 avgusta 2017 goda. Tiumen-Tobolsk: Tiumenskii gosudarstvennyi universitet, pp. 183-185. (in Russian)

164. Sulkarnaeva L.D., 2018. The current state of knowledge of urban ecosystem services in Russia, in: Ekonomicheskii koridor "Kitai–Mongoliia–Rossiia": geograficheskie i ekologicheskie faktory i vozmozhnosti territorialnogo razvitiia : Tezisy Mezhdunarodnoi geograficheskoi konferentsii, Irkutsk, p. Listvianka, 20–26 avgusta 2018 goda. Irkutsk, p. Listvianka: Institut geografii im. V.B. Sochavy Sibirskogo otdeleniia Rossiiskoi akademii nauk, pp. 240-241. (in Russian)

165. Sulkarnaeva, L., 2018. The current state of the art of urban ecosystem services assessment in Russia, in: Practical Geography and XXI Century Challenges : International Geographical Union Thematic Conference dedicated to the Centennial of the Institute of Geography of the Russian Academy of Sciences, Moscow, Vol. Part 1. Moscow: Geography Institute of RAS, pp. 311-315.

166. Sulkarnaeva, L.D., 2018. Feasibility of ecosystem services concept implementation for Russian Cities. Journal of Environmental Management and Tourism 9, 7(31). DOI 10.14505/jemt.v9.7(31).04.

167. Sulkarnaeva, L., Marinskikh, D., 2019. Experience in assessing Tyumen ecosystem services, in: Biodiversity and Ecosystem Services – Management Principles in the Russia and International Processes: International Conference (Moscow, November 19–20, 2019). Moscow: Biodiversity Conservation Center Publishers, pp. 59-61.

168. Sulkarnaeva, L., 2017. The current state of the art of urban ecosystem services assessment in Russia, in: Landscape science: theory, methods, landscape-ecological support of land use and sustainable development, pp. 191-193. (in Russian)

169. Sulkarnaeva, L.D., 2019. The Problems and Prospects of Urban Ecosystem Services Assessment in Russia, in: Urbanization and Regional Development in Russia and Europe : The Book of Abstracts and Program of 2nd International Scientific Conference. Москва: Geography Institute of RAS, pp. 71.

170. Suprunenko, Z.A., Martynova, N.V., 2012. Creation of comfortable environment for person in conditions of urbanization. Novye idei novogo veka: materialy mezhdunarodnoi nauchnoi konferentsii FAD TOGU 2, 111-115. (in Russian)

171. Surkov, N.A., Iurlova, V.A., 2013. Ekonomicheskaia otsenka pochvozashchitnoi funktsii lesa. Innovatsii i investitsii 3, 176-178. (in Russian)

172. Suslov, A.V., Nagimov, Z.YA., Korelina, A.A., 2021. Organization of monitoring of plantings in forest parks of the city of Yekaterinburg with the use of mathematical and statistical methods. Uspekhi sovremennogo estestvoznaniia 6, 35-41. DOI 10.17513/use.37638 (in Russian)

173. Tarasova, N.P., Bednova, O.V., Kuznetsov, V.A, 2011. A system of urban conservation areas, and sustainable development of a megalopolis. Ekologiia urbanizirovannykh territorii 3, 12-17. (in Russian)

174. Targaeva, E.E., Andreeva, O.S., 2018. Formation of the ecological framework of the industrial city: specific features (a case study of Novokuznetsk). Geograficheskii vestnik 3(46), 83-91. DOI 10.17072/2079-7877-2018-3-83-91 (in Russian)

175. Terekhova, V.A., 2015. Bioindication and biotesting as the basis of triad approach by ranking the quality of soil, in: Rol pochv v biosfere i zhizni cheloveka : Mezhdunarodnaia nauchnaia konferentsiia: K 100-letiiu so dnia rozhdeniia akademika G.V. Dobrovolskogo, k Mezhdunarodnomu godu pochv. Moskva: OOO "MAKS Press", pp. 248-249. (in Russian)

176. Tikhonova, I.O., Markelova, S.A., 2017. The applicability of ecosystem services for urban water objects, in: Ekologicheskie problemy promyshlennykh gorodov : sbornik nauchnykh trudov po materialam 8-i Mezhdunarodnoi nauchno-prakticheskoi konferentsii. – Saratov: Saratovskii gosudarstvennyi tekhnicheskii universitet imeni Gagarina Iu.A., pp. 78-82. (in Russian)

177. Tikhonova, I.O., 2018. Water resources management in the city basins - ecosystem services and functions, in: Ekologiia rechnykh basseinov : Trudy IX Mezhdunarodnoi nauchno-

prakticheskoi konferentsii, Suzdal, 05–08 sentiabria 2018 goda. Suzdal: Vladimirskii gosudarstvennyi universitet im. Aleksandra Grigorevicha i Nikolaia Grigorevicha Stoletovykh, pp. 646-650. (in Russian)

178. Titova, G.D., 2016. The ecosystem as a control object. Vestnik Sankt-Peterburgskogo Universiteta, Seriya Geologiya i Geografiya 1, 59-72.

179. Tregubov, O.V., Kochergina, M.V., Furmenkova, E.S., Pripoltseva, A.S., 2014. Species diversity and state of stands in the northern greenbelt district of the city of Voronezh. Lesotekhnicheskii zhurnal 4, 3(15), 61-76. DOI 10.12737/6270 (in Russian)

180. Tsibulnikova, M.R., Pospelova, A.A., 2011. Significance of ecosystem services economic estimation for conservation and efficient use of natural landscapes. Vestnik Tomskogo gosudarstvennogo universiteta 351, 187-193. (In Russian)

181. Umarova, M.Z., Ashurbekova, T.N., 2015. Ekologicheskie problemy i zabolevaemost naseleniia goroda Groznyi zlokachestvennymi novoobrazovaniiami. Problemy razvitiia APK regiona 22, 2(22), 64-69. (in Russian)

182. Van-Kho-Bin, E., Kazantsev, P., 2021. Natural framework design theory and practice in urban planning. Arkhitektura i dizain: istoriia, teoriia, innovatsii 5, 264-270. (in Russian)

183. Vasenev, V.I., Dovletyarova, E., Cheng, Z., Prokof’eva, T., Morel, J., Ananyeva, N., 2019. SUITMA 9: Urbanization as a Challenge and an Opportunity for Soils Functions and Ecosystem Services, in: Vasenev, V., Dovletyarova, E., Cheng, Z., Prokof’eva, T., Morel, J., Ananyeva, N. (eds) Urbanization: Challenge and Opportunity for Soil Functions and Ecosystem Services. SUITMA 2017. Springer Geography. Springer, Cham. https://doi.org/10.1007/978-3-319-89602-1_1

184. Vasenev, V.I., Dovletyarova, E.A., Hajiaghayeva, R.A., Plyushchikov, V.G., Cheng, Z., Stoorvogel, J.J., 2018. MEGACITIES 2050: From urbanization risks towards sustainable urban development, in: Megacities 2050: Environmental Consequences of Urbanization, Moscow, September 12-14, 2018. Cham: Springer International Publishing AG, pp. 3-5. DOI 10.1007/978-3-319-70557-6_1.

185. Vasenev, V.I., Dovletyarova, E.A., Plyushchikov, V.G., Valentini R., 2016. Megacities 2050: from Urbanization Threats to Sustainable Urban Development. RUDN Journal of Agronomy and Animal Industries 4, 7-9. DOI 10.22363/2312-797X-2016-4-7-9.

186. Vasenev, V., Dovletyarova, E., Veretelnikova, I., Fatiev, M., Valentini, R., Calfapietra, C., Cheng, Z., 2020. Smart and Sustainable Cities: From Environmental Threats Towards Nature Based Solutions and Sustainable Management, in: Vasenev, V., Dovletyarova, E., Cheng, Z., Valentini, R., Calfapietra, C. (eds) Green Technologies and Infrastructure to Enhance Urban Ecosystem Services. SSC 2018. Cham. Springer Geography. Springer, pp.1-3. https://doi.org/10.1007/978-3-030-16091-3_1

187. Vasenev, V.I., Fatiev, M.M., Lakeev, P.S., Mazirov, I.M., Trubina, A.E., Vasenev, I.I., Valentini, R., Methodology of functional-ecological monitoring of objects and landscaping of Moscow. Vestnik Rossiiskogo universiteta druzhby narodov. Seriia: Agronomiia i zhivotnovodstvo 5, 15-27. (in Russian)

188. Vasenev, V.I., Makarov, O.A., Ananyeva, N.D., 2012. Specific features of the ecological functioning of urban soils in Moscow and Moscow oblast. Eurasian Soil Science 45(2), 194-205.

189. Vasenev, V.I., Van Oudenhoven, A.P.E., Romzaykina, O.N., Hajiaghaeva, R.A., 2018. The Ecological Functions and Ecosystem Services of Urban and Technogenic Soils: from Theory to Practice (A Review). Eurasian Soil Science 51, 1119–1132. doi:10.1134/s1064229318100137

190. Vetrova, I.N., 2006. Evaluation of ecological functions of greenery system in the city Stavropol. Ekologicheskii Vestnik Severnogo Kavkaza 2(1), 105-109. (in Russian)

191. Vizirskaia, M.M., Tikhonova, M.V., Shchepeleva, A.S., Mazirov, I.M., 2015. Ekologicheskaia otsenka funktsii regulirovaniia sostava atmosfernogo vozdukha podzolistykh pochv lesnykh ekosistem v usloviiakh Moskovskogo megapolisa (na primere LOD RGAU-MSKhA). Aktualnye problemy gumanitarnykh i estestvennykh nauk 9-2, 224-231. (in Russian)

192. Vorobeva, I.B., 2018. Ecological functions of territories with various degree of urbanization under conditions of eastern Siberia. Uspekhi sovremennogo estestvoznaniia 10, 96-101. (in Russian)
193. Udina, N.V., Lysova, E.P., Paramonova, O.N., 2020. Development of methodological approach to formation and selection of the “green belt" while ensuring environmental safety of the cities. Inzhenernyi vestnik Dona 4(64), 6424. (in Russian)
194. Zakharov, K.V., 2018. The attitude citizens to wildlife: objective choice or subjective wishes. Biulleten Moskovskogo obshchestva ispytatelei prirody. Otdel biologicheskii 123(6), 3-11. (in Russian)
195. Zakharov, K.V., Medvedkov, A.A., Ivanova, E.Yu., 2019. Technology of geoecological assessment of urbanized territories(on the example of the near Moscow region). InterCarto. InterGIS 25(1), 352-361. DOI 10.35595/2414-9179-2019-1-25-362-373 (in Russian)
196. Zhadobin, A.V., Kazeev, K.SH., Lesina, A., Kazeev, D.K., Kolesnikov, S.I., 2019. Assessment of the ecological condition of soils in Rostov Zoo. Vestnik Permskogo natsionalnogo issledovatelskogo politekhnicheskogo universiteta. Prikladnaia ekologiia. Urbanistika 1(33), 131-141. DOI 10.15593/2409-5125/2019.01.09 (in Russian)
197. Zubkova, T., Kavtaradze, D., 2019. Soils of urban ecosystems, environmental and social risks, in: Biodiversity and Ecosystem Services – Management Principles in the Russia and International Processes: International Conference (Moscow, November 19–20, 2019). Moscow: Biodiversity Conservation Center Publishers, pp. 74-75.

Supplementary material S3. List of papers used for content analysis

No	Source
1.	Жадобин, А. В., Казеев, К. Ш., Лесина, А. Л., Александров, А. А., Казеев, Д. К., & Колесников, С. И. (2019). Оценка экологического состояния почв Ростовского зоопарка. Вестник Пермского национального исследовательского политехнического университета. Прикладная экология. Урбанистика, (1), 131-141.<br><br>Zhadobin, A.V., Kazeev, K.SH., Lesina, A., Kazeev, D.K., Kolesnikov, S.I., 2019. Assessment of the ecological condition of soils in Rostov Zoo. Vestnik Permskogo natsionalnogo issledovatelskogo politekhnicheskogo universiteta. Prikladnaia ekologiia. Urbanistika 1(33), 131-141. DOI 10.15593/2409-5125/2019.01.09 (in Russian)
2.	Дубенок, Н. Н., Кузьмичев, В. В., & Лебедев, А. В. (2018). Рост и продуктивность древостоев сосны и лиственницы в условиях городской среды. Вестник Поволжского государственного технологического университета. Серия: Лес. Экология. Природопользование, 1 (37), 54-71.<br><br>Dubenok, N.N., Kuzmichev, V.V., Lebedev, A.V., 2018. Growth and productivity of pine and larch stands under conditions of urban environment. Vestnik Povolzhskogo gosudarstvennogo tekhnologicheskogo universiteta. Seriia: Les. Ekologiia. Prirodopolzovanie 1(37), 54-71. DOI 10.15350/2306-2827.2018.1.54 (in Russian)
3.	Душкова, Д. О., & Кириллов, С. Н. (2016). Зеленая инфраструктура города: опыт Германии. Вестник Волгоградского государственного университета. Серия 3: Экономика. Экология, (2 (35)), 136-147.<br><br>Dushkova, D.O., Kirillov, S.N., 2016. Urban green infrastructure: German experience. Vestnik Volgogradskogo gosudarstvennogo universiteta. Seriia 3: Ekonomika. Ekologiia 2(35), 136-147. DOI 10.15688/jvolsu3.2016.2.14 (in Russian)
4.	Лисина, Н. Л. (2015). Современное состояние и проблемы окружающей среды в городах. Вестник Кемеровского государственного университета, (4-2 (64)), 237-241. 2015 (https://elibrary.ru/item.asp?id=24986458)<br><br>Lisina, N.L., 2015. Current status and problems of the urban environment. Vestnik Kemerovskogo gosudarstvennogo universiteta 4-2(64), 242-246. (in Russian)
5.	Катаева, Ю. В., & Лапин, А. В. (2014). Формирование методического подхода к интегральной оценке качества городской среды. Вестник пермского университета. Серия: экономика, (2), 31-39.<br><br>Kataeva, Y.V., Lapin, A.V., 2014. Developing of methodical approach to urban space integrated estimation. Vestnik Permskogo universiteta. Seriia: Ekonomika 2(21), 31-39. (in Russian)
6.	Трегубов, О. В., Кочергина, М. В., Фурменкова, Е. С., & Припольцева, А. С. (2014). Видовое разнообразие и состояние насаждений лесопарковой зоны в Северном микрорайоне городского округа город Воронеж. Лесотехнический журнал, 4(3 (15)), 61-76.<br><br>Tregubov, O.V., Kochergina, M.V., Furmenkova, E.S., Pripoltseva, A.S., 2014. Species diversity and state of stands in the northern greenbelt district of the city of Voronezh. Lesotekhnicheskii zhurnal 4, 3(15), 61-76. DOI 10.12737/6270 (in Russian)
7.	Васенев, В. И., Ананьева, Н. Д., & Макаров, О. А. (2012). Особенности экологического функционирования конструктоземов на территории Москвы и Московской области. Почвоведение, (2), 224-224.<br><br>Vasenev, V.I., Makarov, O.A., Ananyeva, N.D., 2012. Specific features of the ecological functioning of urban soils in Moscow and Moscow oblast. Eurasian Soil Science 45(2), 194-205.
8.	Pashkevich, M. A., Bech, J., Matveeva, V. A., & Alekseenko, A. V. (2020). Biogeochemical assessment of soils and plants in industrial, residential and recreational areas of Saint Petersburg. Записки Горного института, 241, 125-130.<br><br>Pashkevich, M.A., Bech, Ja., Matveeva, V.A., Alekseenko A.V., 2020. Biogeochemical assessment of soils and plants in industrial, residential and recreational areas of Saint Petersburg. Journal of Mining Institute 241, 1, 125-130. DOI 10.31897/PMI.2020.1.125
9.	Russo, A., Cirella G.T., 2018. Modern compact cities: How much greenery do we need? International Journal of Environmental Research and Public Health 15, 10, 2180. DOI 10.3390/ijerph15102180
10.	Russo, A., Escobedo, F.J., Cirella, G.T., Zerbe S., 2017. Edible green infrastructure: An approach and review of provisioning ecosystem services and disservices in urban environments. Agriculture, Ecosystems & Environment 242, 53-66. DOI 10.1016/j.agee.2017.03.026
11.	Matasov, V., Belelli Marchesini, L., Yaroslavtsev, A., Sala, G., Fareeva, O., Seregin, I., Castaldi, S., Vasenev, V., Valentini, R., 2020. IoT Monitoring of Urban Tree Ecosystem Services: Possibilities and Challenges. Forests 11, 775. doi:10.3390/f11070775
12.	Ignatieva, M., Haase, D., Dushkova, D., Haase, A., 2020. Lawns in Cities: From a Globalised Urban Green Space Phenomenon to Sustainable Nature-Based Solutions. Land 9, 73. doi:10.3390/land9030073
13.	Климанова, О. А., Колбовский, Е. Ю., & Илларионова, О. А. (2018). Экологический каркас крупнейших городов Российской Федерации: современная структура, территориальное планирование и проблемы развития. Вестник Санкт-Петербургского университета. Науки о Земле, 63(2), 127-146.

No	Source
	Klimanova, O.A., Kolbowsky, E.Yu., Illarionova, O.A., 2018. The ecological framework of Russian major cities: Spatial structure, territorial planning and main problems of development. Vestnik of Saint Petersburg University. Earth Sciences 63(2), 127-146. DOI 10.21638/11701/spbu07.2018.201.
14.	Бобылев, С. Н., & Порфирьев, Б. Н. (2016). Устойчивое развитие крупнейших городов и мегаполисов: фактор экосистемных услуг. Вестник Московского университета. Серия 6. Экономика, (6), 3-21.<br><br>Bobylev, S.N., Porfiriev, B.N., 2016. Sustainable development of largest cities and megalopolises: a factor of ecosystem services. Vestnik Moskovskogo universiteta. Seriia 6: Ekonomika 6, 3-21. (in Russian)
15.	Цибульникова, М. Р., & Поспелова, А. А. (2011). Значение экономической оценки экосистемных услуг для сохранения и рационального использования природных ландшафтов. Вестник Томского государственного университета, (351), 187-193.<br><br>Tsibulnikova, M.R., Pospelova, A.A., 2011. Significance of ecosystem services economic estimation for conservation and efficient use of natural landscapes. Vestnik Tomskogo gosudarstvennogo universiteta 351, 187-193. (In Russian)
16.	Морозова, Г. Ю., & Дебелая, И. Д. (2018). Зеленая инфраструктура как фактор обеспечения устойчивого развития Хабаровска. Экономика региона, 14(2), 562-574.<br><br>Morozova, G.Yu., Debelaya, I.D., 2018. Green Infrastructure as a Factor for Sustainable Development of Khabarovsk. Economy of Region 14(2), 562-574. DOI 10.17059/2018-2-18.
